# Studies on the Assembly Characteristics of Large Subunit Ribosomal Proteins in *S. cerevisae*

**DOI:** 10.1371/journal.pone.0068412

**Published:** 2013-07-10

**Authors:** Uli Ohmayer, Michael Gamalinda, Martina Sauert, Julius Ossowski, Gisela Pöll, Jan Linnemann, Thomas Hierlmeier, Jorge Perez-Fernandez, Beril Kumcuoglu, Isabelle Leger-Silvestre, Marlène Faubladier, Joachim Griesenbeck, John Woolford, Herbert Tschochner, Philipp Milkereit

**Affiliations:** 1 Lehrstuhl für Biochemie III, Universität Regensburg, Regensburg, Germany; 2 Department of Biological Sciences, Carnegie Mellon University, Pittsburgh, Pennsylvania, United States of America; 3 Laboratoire de Biologie Moléculaire Eucaryote, UMR 5099, Universite Paul Sabatier, Toulouse, France; University of Louisville, United States of America

## Abstract

During the assembly process of ribosomal subunits, their structural components, the ribosomal RNAs (rRNAs) and the ribosomal proteins (r-proteins) have to join together in a highly dynamic and defined manner to enable the efficient formation of functional ribosomes. In this work, the assembly of large ribosomal subunit (LSU) r-proteins from the eukaryote *S. cerevisiae* was systematically investigated. Groups of LSU r-proteins with specific assembly characteristics were detected by comparing the protein composition of affinity purified early, middle, late or mature LSU (precursor) particles by semi-quantitative mass spectrometry. The impact of yeast LSU r-proteins rpL25, rpL2, rpL43, and rpL21 on the composition of intermediate to late nuclear LSU precursors was analyzed in more detail. Effects of these proteins on the assembly states of other r-proteins and on the transient LSU precursor association of several ribosome biogenesis factors, including Nog2, Rsa4 and Nop53, are discussed.

## Introduction

Ribosomes catalyze the translation of mRNA into proteins in all living cells. They are composed of a small and of a large ribosomal subunit (SSU and LSU, respectively) which are made in eukaryotic organisms of four ribosomal RNAs (rRNA) and more than 75 ribosomal proteins (r-proteins) [1]. Recent atomic resolution structure models of prokaryotic and eukaryotic ribosomes showed the sophisticated three-dimensional organization of the ribosomal components in this large ribonucleoprotein (RNP) complex [2–9]. To produce a high quantity of ribosomes in dividing cells, a substantial amount of resources has to be spent to synthesize rRNAs and r-proteins [10]. In addition during ribosome maturation, the nascent rRNAs are modified, processed, folded, assembled with r-proteins, and, in eukaryotic cells, transported from the nucleolus to the cytoplasm. The processing of eukaryotic precursor rRNAs, their modification, and the intracellular transport of eukaryotic pre-ribosomes were investigated in more detail in *S. cerevisiae* (in the following called yeast) and in mammalian cells (reviewed in 11).

Much of our knowledge on how r-proteins and rRNAs assemble comes from studies of ribosomes of the prokaryote *E. coli in vitro*. Functional ribosomal subunits of *E. coli* can be reconstituted by bringing together purified rRNAs and r-proteins of a ribosomal subunit in appropriate buffer- and temperature conditions (see 12,13 for reviews). In depth characterization of the factor-free *in vitro* assembly of ribosomal subunits showed that efficient binding of individual r-proteins to rRNA often requires the previous binding of certain other r-proteins. Accordingly, r-proteins were classified as primary, secondary, or tertiary *in vitro* binders depending on whether they stably associate with rRNA in the absence, or only in the presence of one other, or more than one other r-protein. In general, time resolved analyses of the process showed a good correlation between the assembly kinetics of SSU r-proteins and their role as primary, secondary or tertiary binders [14]. Kinetic RNA structure probing experiments indicated that the mode of interaction of individual r-proteins with rRNA can change during the time course of the *in vitro* reconstitution procedure: individual r-proteins often contact different regions of rRNA in mature ribosomes and some of these rRNA contacts are established late during assembly in the *in vitro* reconstitution experiments [15,16].

*In vivo*, the interplay of all the processes involved in maturation of ribosomes might modulate how robust interactions between r-proteins and rRNAs are formed. For example, initial interactions between r-proteins and rRNAs might already occur *in vivo* during ongoing synthesis of rRNA ( [17], see 18 for critical discussion). Moreover, the presence and removal of spacer sequences in precursor rRNAs and the modification of rRNA during ribosome maturation might impact r-protein assembly events [19]. In addition, in eukaryotes, a large number of non-ribosomal proteins and small non-coding RNAs associate *in vivo* in a dynamic way with ribosomal precursor particles (reviewed in 11,20–23). Most of these factors are required for efficient production of either one of the ribosomal subunits *in vivo*. Clear evidence for functional interrelationships between the activities of a number of ribosome biogenesis factors and r-protein assembly events were provided [24–35]. Finally, in eukaryotes most steps of ribosomal maturation take place in the nucleolus where precursor rRNAs are synthesized, but final maturation of newly made ribosomal subunits only occurs after their nuclear export to the cytoplasm (reviewed in 36). Some assembly events might be affected by the prevalent nuclear or cytoplasmic localization of biogenesis factors or of free pools of one or the other r-protein.

The kinetics of *in vivo* assembly of eukaryotic LSU r-proteins was studied about three decades ago by several groups. Newly made cellular proteins were metabolically labeled for various times, and subsequently mature ribosomes were isolated from cellular extracts. LSU r-proteins showing comparably high labeling in isolated ribosomes after short metabolic pulse times could be interpreted as being incorporated late during ribosome maturation into ribosomal precursors. Unambiguous detection of some of the r-proteins was difficult in these studies since two dimensional gel electrophoresis systems with varying properties in the resolution of r-proteins were used for their identification (for interpretation of nomenclatures used in these studies see [37–39], please note that in this manuscript, if not stated otherwise in the text, eukaryotic r-proteins are named according to the yeast standard nomenclature [37,40]). Nevertheless the groups of Tavitian, McConkey and Planta consistently found evidence for rpL10 and rpL24 belonging to the group of r-proteins being incorporated last during eukaryotic large ribosomal subunit maturation in mouse, human and yeast cells, respectively [41–44]. This notion was further supported by experiments aiming to identify LSU r-proteins specifically absent from nuclear LSU precursors which contained detectable amounts of most other LSU r-proteins [41,42,44]. Apart from that, a number of less consistent candidates for late assembling LSU r-proteins were found by these approaches. Among them were rpL29 and rpL40 [41], rpL19 [42,44], rpP2 [42] and several r-proteins of unclear identity. Several groups have taken a step further and analyzed the ability of free pools of specific eukaryotic LSU r-proteins to replace their respective copies in mature ribosomes in conditions in which neo-production of ribosomes is thought to be inhibited. Mammalian rpP0, rpP1, rpP2, rpL10, rpL19 and rpL24 [43,45,46] and yeast rpP1, rpP2, rpL10 and, possibly rpP0 [39,47–50] were identified as candidates for exchangeable LSU r-proteins in these studies. Interestingly, there is evidence that yeast rpL10 and rpP1 and rpP2 are underrepresented in 60S ribosomal subunits not actively engaged in mRNA translation, opening up the possibility that their exchange and / or assembly might be regulated during the ribosomal translation cycle [50–52].

More recently, the composition of yeast LSU precursor particles affinity purified via associated tagged ribosome biogenesis factors was characterized in a number of studies (reviewed in 11,21–23). These analyses mainly focused on the detection of the LSU rRNA precursors and ribosome biogenesis factors present in these particles. In part, characterization of the r-protein content was hampered due to some amounts of contaminating mature ribosomal subunits in the affinity purified precursor particle fractions, especially when only qualitative methodology could be applied for their identification. Nevertheless these approaches provided further evidence that the exchangeable yeast LSU r-proteins, rpP1, rpP2, and rpL10, together with rpP0, are at least partially depleted in affinity purified preparations of LSU precursor particles [25–27,53–55]. Incorporation of a subset of LSU r-proteins into specific ribosomal precursor particle populations was also directly tested by affinity purification of tagged r-proteins from cellular extracts and subsequent detection of associated rRNA precursors. These studies indicated that the final incorporation of rpL10 [56] and rpL40 [57] into LSUs is established only after most of the processing steps of LSU rRNA precursors are accomplished. For rpL5, rpL11 [31] and rpL35 [58] association was detected already with early, and for rpL26 [59], rpL17 and rpL37 [30,32] association was seen mainly starting at the level of intermediate nuclear LSU precursor populations.

Quantitative mass spectrometric approaches were previously used successfully to detect changes in the biogenesis factor compositions of various ribosomal precursors isolated from wildtype or mutant yeast strains [33,60–62]. Moreover, evidence for changes in the r-protein composition of early nuclear LSU intermediates in mutants of r-protein and biogenesis factor genes could be obtained by this approach [29,32]. In the present work, the assembly characteristics of yeast LSU r-proteins and biogenesis factors were analyzed by comparing their respective amounts in affinity purified mature LSUs and LSU precursors using quantitative mass spectrometry. Previous work showed that expression shut down of most of the yeast r-proteins leads to specific ribosome maturation phenotypes and to severe growth defects [58,63–70]. In this study, we focused on mutants of RPL2, RPL43, RPL25 and RPL21, in which LSU precursors were partially destabilized and successive intermediate to late nuclear steps in LSU maturation were impeded [65,70]. A large dataset was generated and statistical analyses were performed to monitor changes in the composition of various LSU particles isolated from wild type or mutant yeast cells in which r-protein genes can be conditionally expressed. We discuss the assembly characteristics of yeast LSU r-proteins as deduced from the data obtained in this work with regard to related previous *in vitro* and *in vivo* analyses.

## Results

### LSU (precursor) particles compared in this study can be grouped into different classes according to their (pre-) rRNA content

**Figure 1:**
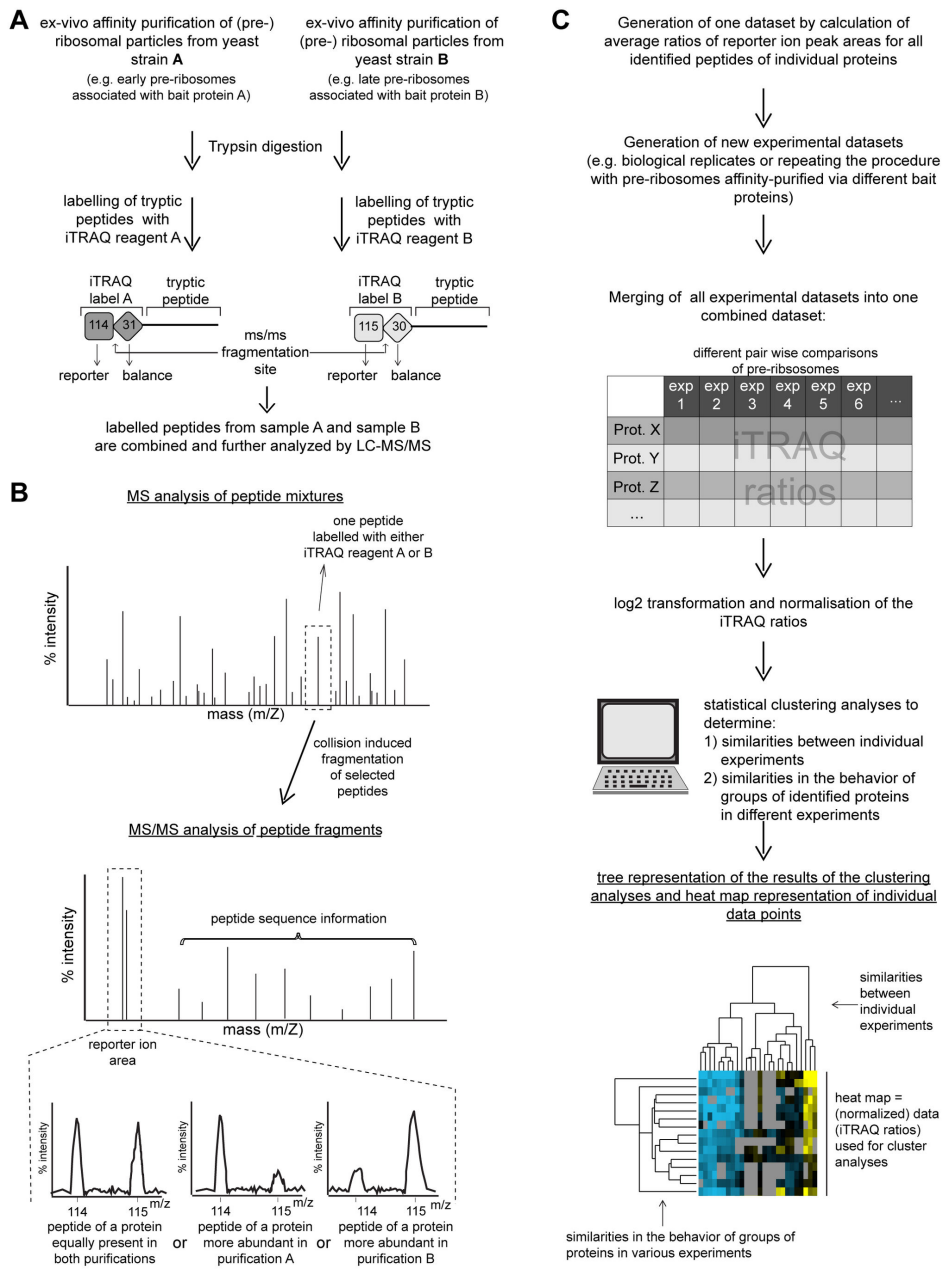
Schematic illustration of the workflow used to characterize the assembly states of (pre-)ribosomal particles. In (**A**) and (**B**) an overview is given on the approach used to purify various (pre-)ribosomal particles from yeast cells and to characterize their protein composition by LC-MS/MS. (**C**) summarizes how the obtained data were further evaluated and visualized.

**Figure 2:**
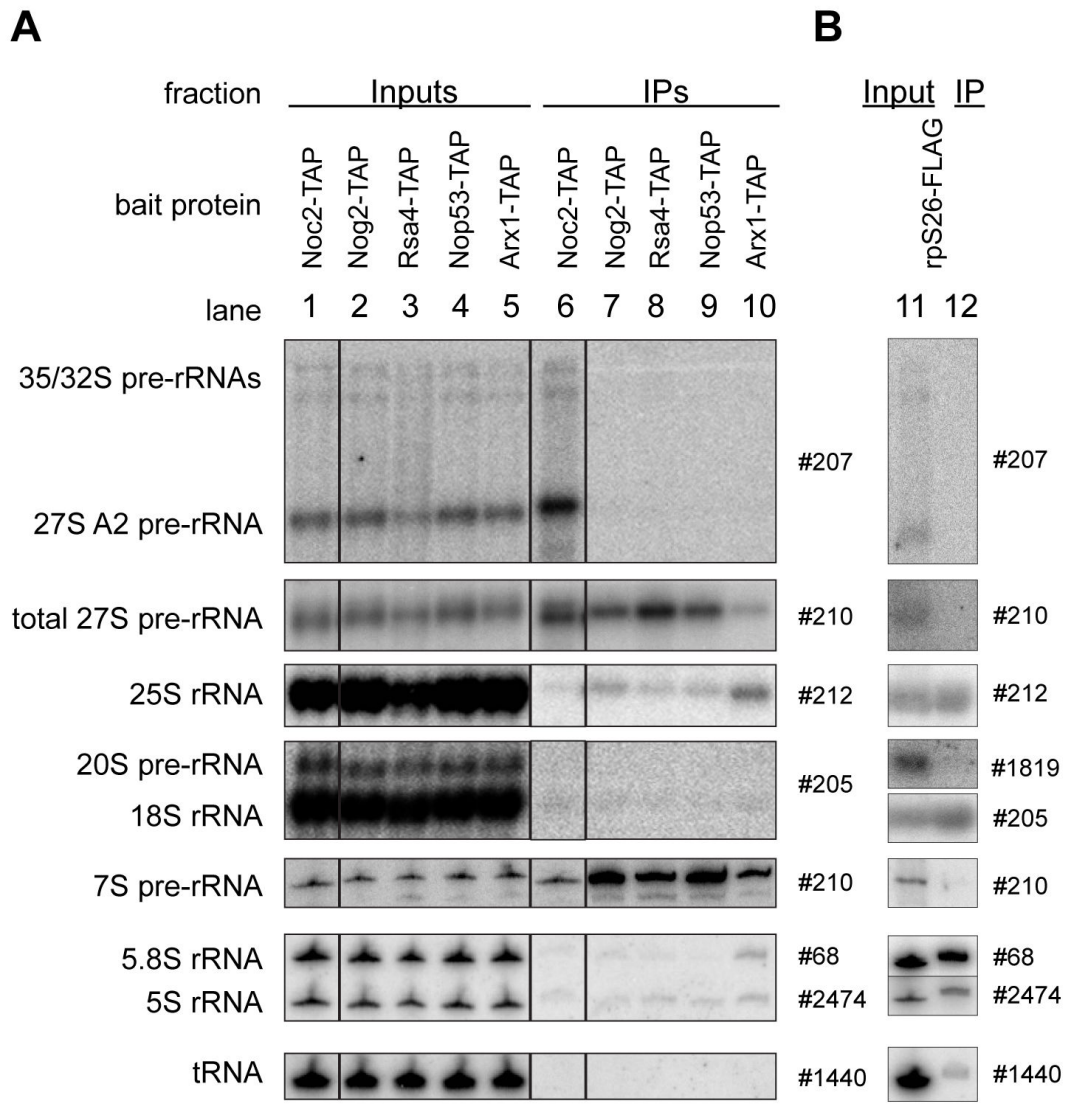
Analysis of the (pre-) rRNAs contained in various affinity purified LSU (precursor) particles. Yeast strains Y1878, Y1879, Y2398, Y2404, Y2410 and Y485 expressing the indicated TAP-tag fusion proteins (**A**) or carrying a C-terminally FLAG-tagged allele of RPS26 under control of the galactose-inducible GAL1/10 promoter (**B**) were cultivated to exponential growth phase in galactose-containing medium (YPG). The tagged proteins were affinity purified from total cellular extracts using rabbit IgG coupled to magnetic beads (**A**) or an anti FLAG M2 matrix (**B**) as described in the materials and methods. (pre-) rRNA species from total cellular extracts (Input) and affinity purified fractions (IP) were analyzed by northern blotting using oligonucleotide probes indicated on the right. Equal signal intensities of the Input and bead (IP) fractions indicate 3% (**A**) or 6% (**B**) co-purification efficiences of the respective (pre-) rRNA with bait proteins.

In order to identify previously unresolved changes in the assembly states of yeast LSU r-proteins, we aimed to compare the protein content of LSU precursor particles of different maturation states to each other and to mature 80S ribosomes by semi-quantitative mass spectrometry. We reasoned that stabilized incorporation of LSU r-proteins or ribosome biogenesis factors into pre-ribosomes at either earlier or later stages of ribosome maturation should be detectable by this comparative approach (see Figure 1 for an overview on the experimental strategy). As a starting point, the maturation states of LSU (precursor) particles used in this study were characterized by analyzing their (pre-) rRNA content. Mature 80S ribosomes were affinity purified *ex-vivo* using tagged rpS26, an apparently late assembling SSU r-protein [71,72]. LSU precursor particles were affinity purified from yeast cellular extracts via several tagged LSU biogenesis factors that interact with pre-ribosomes during different maturation stages. The bait proteins used included chromosomally encoded C-terminally TAP tagged pre-60S biogenesis factors Noc2, Nog2, Rsa4, Nop53 and Arx1 which were previously described to bind to either earlier or later LSU precursor populations [53,73–77]. Total RNAs were extracted from respective cellular extracts and from the affinity purified fractions and were analyzed by northern blotting (Figure 2). The purified LSU precursors could be grouped into “early”, “intermediate” and “late” classes according to their (pre-) rRNA content (Figure S1 gives an overview on the yeast LSU pre-rRNA processing pathway). Noc2-TAP predominantly co-purified early to intermediate LSU precursor particles containing 27SA and 27SB LSU pre-rRNAs (Figure 2, compare lane 1 and lane 6, see also 73). Consistent with previous results, Nog2-TAP, Rsa4-TAP and Nop53-TAP co-purified mainly intermediate to late LSU precursors containing 27SB pre-rRNA and large amounts of 7S pre-rRNA (Figure 2, compare lanes 2-4 to lanes 7-9, see also 53,74,75,77). Moreover, co-purification of 25.5/25S rRNA over background levels was detected in these cases (Figure 2, compare 25S rRNA signals in lanes 7-9 with the one in lane 6) further indicating association of these proteins with nascent ribosomes in which separation of 25S rRNA and 5.8S rRNA precursors occurred through cleavage in the internal transcribed spacer 2 (ITS2) pre-rRNA region (see scheme of the LSU pre-rRNA processing pathway in Figure S1). Arx1-TAP predominantly co-purified a late class of (pre-) ribosomal particles containing 7S pre-rRNA, 25.5/25S rRNA and 5.8S rRNA (Figure 2, compare lane 5 to lane 10). 25S rRNA and 5.8S rRNA co-purification with Arx1-TAP could be due to its association with fully processed nascent LSUs [74,78,79] or due to its binding to free mature LSUs liberated after translation termination from SSUs and mRNA ( [80], see discussion in [61]). Finally, analysis of the rRNA co-purifying with rpS26-FLAG confirmed previous observations suggesting its stable incorporation into SSUs after final conversion of 20S SSU rRNA precursors into 18S rRNA occurred (Figure 2, compare 20S pre-rRNA signals and 18S rRNA signals in lanes 11 and 12, see also 71,72).

### The proteomes of the compared LSU (precursor) particles differ in the levels of individual co-purified LSU biogenesis factors

**Figure 3:**
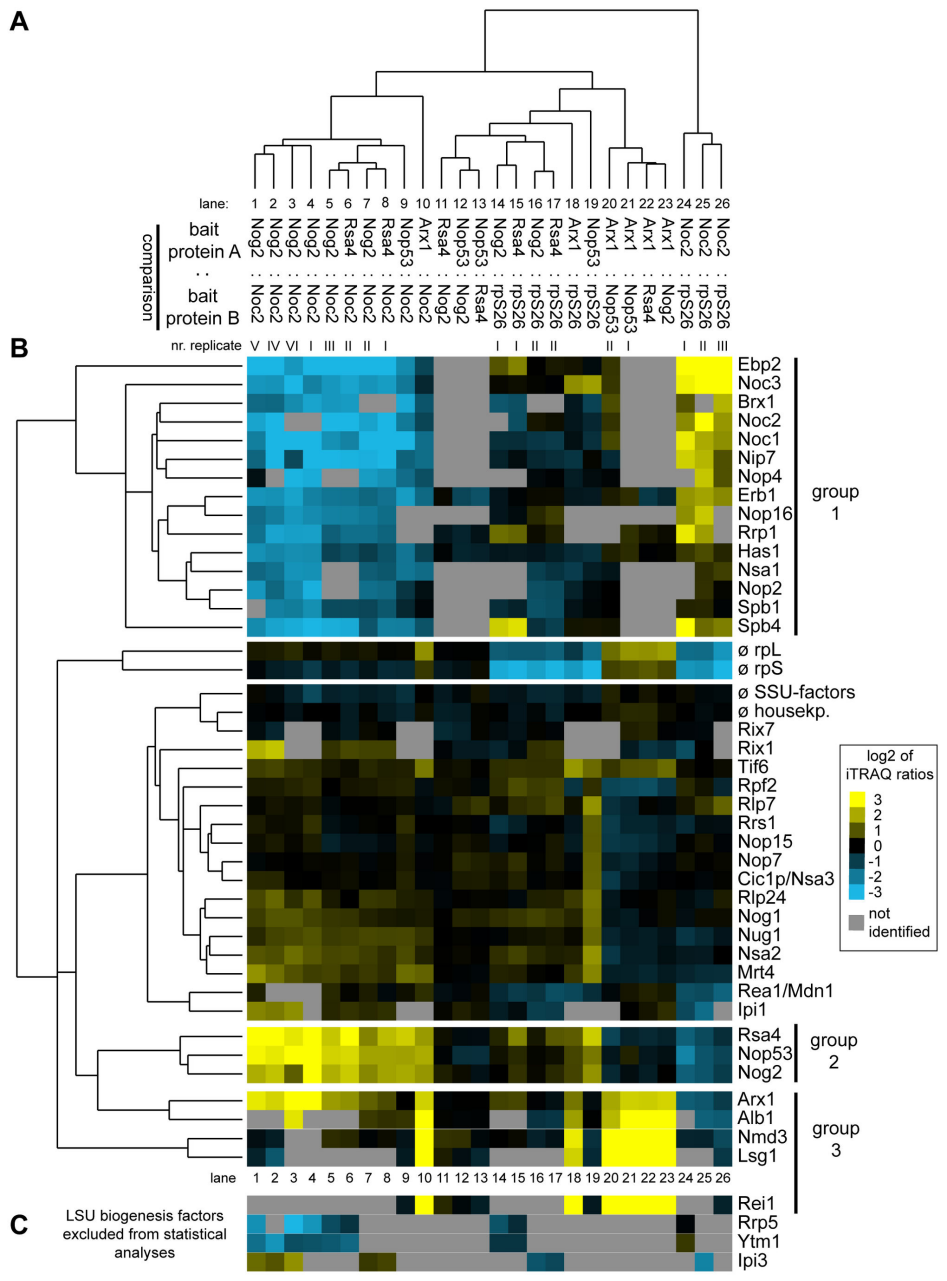
Comparative analyses of the LSU biogenesis factor content in various affinity purified LSU (precursor) particles by semi-quantitative mass spectrometry. (Pre-) ribosomal particles were affinity purified from total cellular extracts of yeast strains Y485, Y1878, Y1879, Y2398, Y2404 and Y2410 expressing FLAG-tagged rpS26 or TAP tagged ribosome biogenesis factors Nog2, Noc2, Rsa4, Nop53 or Arx1. Proteins contained in the affinity purified fractions were compared by semi-quantitative mass spectrometry in the pair wise combinations indicated in (**A**) using iTRAQ reagents as described in Materials and Methods. The resulting iTRAQ ratios of all LSU biogenesis factors that were identified in more than 50% of all pair wise comparisons were combined to one dataset which also included average ratios of housekeeping proteins, SSU biogenesis factors, LSU and SSU r-proteins. This dataset was further analyzed by statistical clustering methods as described in Materials and Methods. In the self-organizing tree shown in (**A**) most similar single comparisons are grouped in the same branches. In the self-organizing tree on the left side of (**B**) the similarity in behavior of the identified LSU biogenesis factors is analyzed. The normalized iTRAQ ratios for each of the proteins measured in the respective pair-wise particle comparison are visualized as a heat map in (**B**). Blue colors represent a low and yellow colors a high average iTRAQ ratio of the respective protein in each comparison (see color code on the right side). A grey color indicates that the respective protein was not identified in the individual experiment shown in this lane. Groups of proteins clustering in one branch which are discussed in more detail in the results part of the manuscript are labeled by bars on the right. In (**C**) the average iTRAQ ratios of a few LSU biogenesis factors are shown which were excluded from the statistical analyses since they were detected in less than 50% of all pair-wise comparisons.

The protein compositions of each of the above described LSU precursor populations and of 80S ribosomes were next compared pair wise by semi quantitative mass spectrometry using iTRAQ reagents. The results from 26 semi-quantitative pair wise comparisons were then combined and normalized into a single dataset that was subjected to hierarchical clustering analyses (see Figure 1 for an overview on the procedure applied, for further details see Materials and Methods). All known LSU biogenesis factors identified in more than 50% of all experiments were included in these statistical analyses. Figure 3A shows the result of this hierarchical clustering analysis addressing the similarity of the 26 different datasets with regard to each other. Biological and technical replicates of particle comparisons of the same type (lane 1-10: intermediate/late particles versus early/intermediate particles; lanes 11-13 intermediate/late particles among each other; lanes 14-19: intermediate/late particles versus 80S ribosomes; lanes 20-23: late versus intermediate/late particles, lanes 24-26: early/intermediate particles versus 80S ribosomes) showed highest similarity to each other and clustered in the same branches (Figure 3A). These results indicated that most of the observed differences in particle composition were reproducible and statistically relevant.

The clustering analyses furthermore revealed that groups of factors had similar characteristics in regard to their association with different LSU precursor particles (Figure 3B). A group of LSU biogenesis factors (group 1) was specifically enriched in pre-ribosomes purified by Noc2-TAP (Figure 3B, lanes 1-10 and 24-26). Besides Noc2, this group consisted of factors such as Noc1, Ebp2, Brx1 and Spb1as well as others for which previous experiments indicated an association with early pre-ribosomes ( [73] and citations therein). A second cluster of LSU biogenesis factors (group 2) arising from these analyses consisted of Nog2, Rsa4 and Nop53 (Figure 3B). Each of these proteins was specifically depleted of early and intermediate pre-LSU populations purified by Noc2-TAP indicating that these proteins mark pre-LSU populations from which Noc2 had already dissociated (Figure 3B, lanes 1-10). Depletion of each of the three proteins was previously shown to result in delay in nuclear processing of 27SB and 7S pre-rRNAs, which are major rRNA precursor components of the pre-ribosomal populations they are associated with ( [53,75,81–83], see also Figure 2 lanes 7-9). A direct comparison of pre-ribosomes purified by Nog2-TAP, Rsa4-TAP or Nop53-TAP indicated that their respective protein (Figure 3B, lanes 11-13) and pre-rRNA (Figure 2, lanes 7-9) composition was highly similar, and largely differed from the one of Noc2-TAP associated pre-ribosomes. Finally, the analyses indicated that another group (group 3) of ribosome biogenesis factors was enriched in (pre-) ribosomal particles purified by Arx1-TAP (Figure 3B, lane 10, 18 and 20-23). This group included Arx1, Alb1, Nmd3, and Lsg1: all of which are thought to be involved in nucleo-cytoplasmic translocation and/or final cytoplasmic maturation steps of yeast LSU precursors [78,79,84–87]. Several other ribosome biogenesis factors were only identified in smaller subsets of the 26 individual experiments and therefore were excluded from statistical analyses. However, when identified, their association behavior was similar to the one of the members of one of the afore mentioned groups (Figure 3C).

### Levels of specific groups of ribosomal proteins are decreased in early and intermediate LSU precursor particles

**Figure 4:**
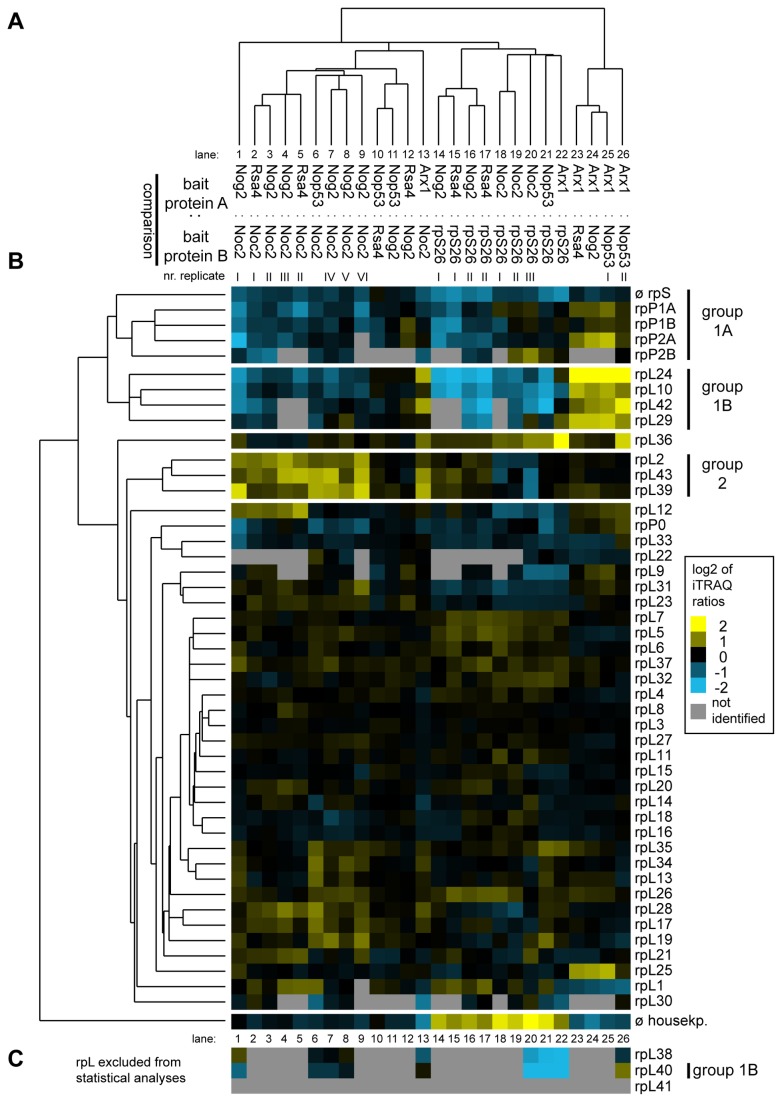
Comparative analyses of the LSU r-protein content in various affinity purified LSU (precursor) particles by semi-quantitative mass spectrometry. The experimental dataset generated as described in Figure 3 was analyzed in regard to iTRAQ ratios of all LSU r-proteins that were identified in more than 50% of all pair wise comparisons. This dataset was further analyzed by statistical clustering methods as described in Materials and Methods. (**A**) shows again a self-organizing tree with most similar single comparisons grouped in the same branches. Accordingly, the similarity in behavior of individual identified LSU r-proteins is shown on the left side of (**B**). The normalized iTRAQ ratios for each protein measured in the respective pair wise particle comparison are visualized as a heat map in (**B**). Blue colors represent a low and yellow colors a high average iTRAQ of each protein in the respective comparison (see color code on the right side). A grey color indicates that the respective protein was not identified in the individual experiment shown in this lane. Groups of proteins clustering in one branch which are discussed in more detail in the results part of the manuscript are labeled on the right in (**B**). (**C**) shows average iTRAQ ratios of the remaining LSU r-proteins that were excluded from the statistical analyses since they were detected in less than 50% of all pair wise comparisons.

Taken together, the previous RNA and protein analyses were in good agreement with the expected compositions of pre-ribosomal populations purified by the chosen bait proteins. Hence, this proves to be a robust approach in systematically characterizing the proteomic composition of various affinity purified complexes. The results of the same 26 semi-quantitative particle comparisons were next analyzed by hierarchical clustering as described above but focusing now on the co-purified large ribosomal subunit proteins (Figure 4). With the exception of r-proteins rpL38, rpL40 and rpL41, all LSU r-proteins could be identified in at least half of all comparisons and thus were included in the analyses. In general, the datasets derived from biological or technical replicates of the same types of comparisons showed the highest similarities among each other (Figure 4A) even if they clustered not always as close to each other as seen in case of the ribosome biogenesis factors (compare general shapes of cluster trees in Figures 3A and 4A and compare for example clustering behaviour of the Nog2: Noc2 dataset I in lane 1 in Figure 4A and in lane 4 in Figure 3A). This indicated that despite the presence of some background levels of mature cytoplasmic 80S ribosomes in all of the purified fractions (Figure 2, faint 18S rRNA signals in lanes 6-10), the prevalent observed differences in relative amounts of individual LSU r-proteins were significant.

One group of large ribosomal subunit proteins (Figure 4B, group 1) was specifically enriched in mature 80S ribosomes and late (pre-)ribosomes purified by rpS26-FLAG and Arx1-TAP, respectively (Figure 4B, lanes 13-26), indicating that stable incorporation of the respective r-proteins into LSU precursors occurs at late stages. The group consisted of the phospho-stalk proteins 1A/B and 2A/B (group 1A) together with rpL24, rpL10, rpL42 and rpL29 (group 1B). Underrepresentation of phospho-stalk proteins rpP0 and rpL12 and a few other r-proteins in (early) LSU precursor particles was seen in several experiments, but was statistically less significant (Figure 4B, lanes 13-26). RpL40, a very small r-protein consisting of only 52 amino acids behaved whenever identified (in less than 50% of all comparisons) very similar to the group of r-proteins consisting of rpL24, rpL10, rpL42, rpL29 and rpP1/2 (Figure 4C, lanes 1, 6-8, 20-22 and 26). Four out of the seven members of this group of LSU r-proteins (rpP1, rpP2, rpL24, rpL29) are encoded by non-essential genes indicating a correlation between their specific assembly behavior and no essential role for LSU production in yeast [66,88–90]. Two of the proteins in this group encoded by essential genes (rpL40 and rpL10) were previously shown to be not strictly required for any of the LSU pre-rRNA processing steps but rather for efficient cytoplasmic accumulation of LSUs [51,57,65].

A second group of LSU r-proteins (Figure 4B, group 2) including rpL2, rpL43 and rpL39, could be distinguished from the other r-proteins due to their specific underrepresentation in early to intermediate pre-ribosomes purified by Noc2-TAP (Figure 4, lanes 1-9 and 13). LSU precursors purified via Noc2-TAP show a low content of 7S pre-rRNA when compared to the ones purified via the other LSU biogenesis factors chosen in this study (Figure 2, lanes 6-10). Accordingly, association of rpL2, rpL39 and rpL43 with LSU precursors might be specifically stabilized after cleavage of 27SB pre-rRNA in the ITS2 region leading to production of 7S pre-rRNA and 25.5S rRNA (Figure S1). rpL2 and rpL43 are encoded by essential genes in yeast and were both described to be required for nuclear processing of 7S pre-rRNA [65].

### General and specific features of the association of LSU r-proteins with LSU precursors addressed by affinity purification of tagged rpL

To analyze in more detail the assembly behavior of LSU r-proteins, several of them were affinity purified *ex vivo* and relative amounts of various co-purifying (pre-) rRNAs were subsequently compared. R-proteins that are stably incorporated at later stages of LSU maturation were expected to co-purify in this type of analyses to a greater extent the late and more mature (pre-) rRNAs than early LSU rRNA precursors.

**Figure 5:**
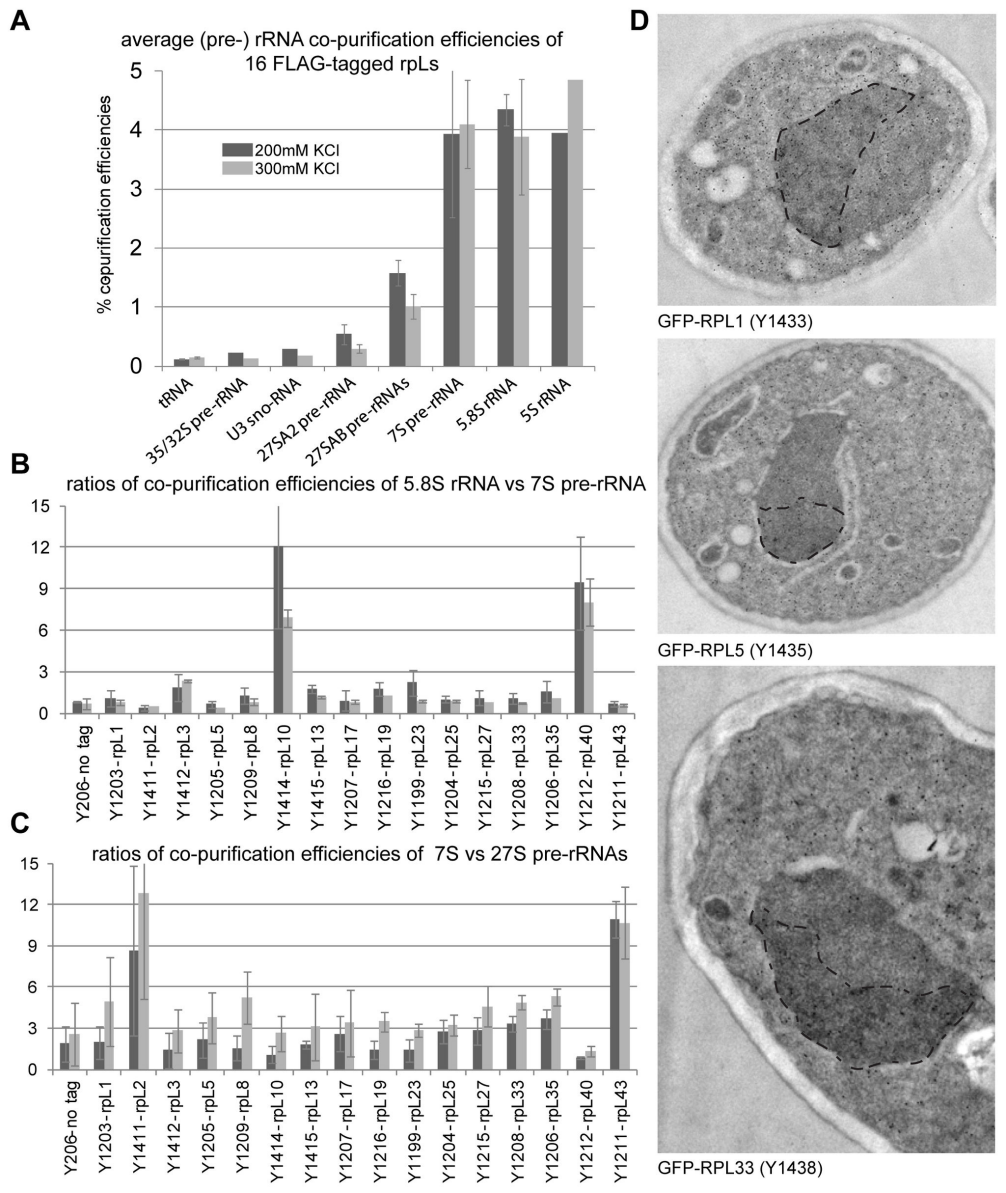
Association of selected tagged LSU r-proteins with LSU precursor particles of different maturation states as indicated by co-purification of LSU (pre-) rRNAs. Total cellular extracts were prepared of 16 yeast strains (indicated in (**B**) and (**C**)) each of which ectopically expressing a FLAG-tagged version of a LSU r-protein complementing the corresponding lethal gene deletion(s) (see Materials and Methods). Tagged proteins were affinity purified on a FLAG affinity matrix as described in Materials and Methods. (Pre-) rRNA species contained in cellular extracts (input fractions) and the corresponding purified fractions were analyzed by northern blotting (Figure S5A and B). In (**A**) the average co-purification efficiencies of the indicated RNAs with the group of analyzed LSU r-proteins were determined by relating the respective signal intensities detected in purified fractions to the ones detected in the extract of a reference yeast strain. In (**B**) is shown the ratio of the efficiency of 5.8S rRNA versus 7S pre-rRNA co-purification for each analyzed LSU r-protein. The ratios of the efficiencies of 7S pre-rRNA versus the 27S pre-rRNA co-purification are shown in (**C**) for each analyzed LSU r-protein. Values obtained when purifications were performed using buffers containing 200 mM or 300 mM potassium chloride (see Materials and Methods) are represented as dark grey or light grey bars, respectively, in (**A**) to (**C**). In (**D**) the green fluorescent protein (GFP) was immuno-detected on sections of chemically fixed yeast cells expressing fusions of GFP with the indicated r-proteins (see Materials and Methods for details). Yeast strains used for the analyses are indicated in brackets. GFP-fusion proteins were labeled with an anti GFP polycolonal antibody recognized by 10nm colloidal gold-conjugated secondary antibodies which are seen in the electron micrographs as black dots. Representative labeled yeast sections are shown. For better visualization the nucleolar regions were manually surrounded by a dashed black line.

Yeast strains that ectopically expressed FLAG tagged variants of selected r-proteins were created to complement the lethal deletions of the corresponding r-protein genes. Cellular extracts of these yeast strains were subjected to affinity purification on an anti-FLAG affinity matrix applying two different salt concentrations. Out of the 16 proteins chosen, rpL10 and rpL40 belonged to the “late” rpL10/rpL40 group (Figure 4B, group 1B), whereas rpL2 and rpL43 were members of the group of LSU r-proteins whose stable assembly seemed to increase concomitant with cleavage in the ITS2 region of pre-rRNA (group 2 in Figure 4B). The co-purified (pre-) rRNA species were analyzed by RNA extraction followed by northern blotting (Figure S5) and the relative efficiencies of purification of individual (pre-) rRNAs were determined by relating the respective quantified signals in affinity purified fractions to the ones in a total cellular extract (Figure 5). On average, LSU r-proteins co-purified the earliest pre-rRNA species (the 35/32S pre-rRNAs) to a comparably weak extent (at levels slightly above background) while further processed pre-rRNA species and rRNAs of mature LSUs were co-purified more efficiently (Figure 5A). This suggests that most of the LSU r-proteins start interacting with pre-ribosomes at early stages with rather low affinity and that binding affinities increase with ongoing LSU maturation. In line with this, the binding to early (32/35S and 27S pre-rRNA containing) LSU precursors could be more easily disrupted by higher salt concentrations than the binding to later pre-ribosomes and mature ribosomes (Figure 5A, compare light grey and dark grey bars). Thus, these data indicated that most of the tested rpLs assemble with LSU precursors in a progressive way during their maturation. The results of previously published work on several members of this large group, namely rpL35 [58], rpL5 [31], rpL7 and rpL8 [29] were in general in agreement with this interpretation. Immuno-detection of three tagged r-proteins in ultrathin sections of yeast cells indicated that in line with previous results [18], assembly of most r-proteins (including the 5S RNP) starts in the nucleolus (Figure 5D). The intranucleolar distribution of tagged r-proteins observed in these experiments did, however, not strictly exclude the occurrence of first assembly events at sites of rDNA transcription (compare with [18]).

Differences in the assembly behavior of individual LSU r-proteins could be detected. For rpL10 and rpL40 (members of the “late” group 1B in Figure 4B), the ratio of 5.8S versus 7S pre-rRNA co-purification efficiency was considerably higher than for all other tested LSU r-proteins (Figure 5B). These observations were in agreement with previous results [53,57] and argued for a pronounced stabilization of the interaction of rpL10 and rpL40 with LSUs after conversion of 7S pre-rRNA into 5.8S rRNA. A further outcome of these analyses was that 7S pre-rRNA co-purified significantly better than 27S pre-rRNA with tagged rpL2 and rpL43 (both members of group 2 in Figure 4B). For none of the other tested LSU r-proteins such a strong change in association with these two subsequent LSU precursors could be detected (Figure 5C). In line with the previous proteome data, these results indicated that rpL2 and rpL43 have a pronounced increase in their binding affinities for LSU precursors after the cleavage in the ITS2 region, leading to the production of 7S pre-rRNA, has occurred. Still, rpL2 and rpL43 clearly co-purified 35/32 and significant levels of 27S pre-rRNA species (Figures S5A & S5B) indicating that they are already recruited to pre-ribosomes of this maturation state, although with lower affinity.

### Changes in the protein composition of LSU precursor particles in selected r-protein gene mutants with intermediate to late nuclear LSU pre-rRNA processing phenotypes

The previous data indicated that nuclear LSU precursors undergo a structural re-organisation which comprises increased binding of the LSU r-proteins of the rpL2/rpL43/rpL39 group, concomitant with cleavage of LSU pre-rRNA in the ITS2 region. RpL2 and rpL43 are both required for conversion of 7S pre-rRNA into 5.8S rRNA which involves partial nuclear trimming of the corresponding ITS2 pre-rRNA region through the exosome [91,92]. Moreover, they bind next to each other in mature LSUs at the subunit interface with many direct interactions among them [2]. rpL39, encoded by a non-essential gene [66,93], sits near the exit tunnel and 5.8S rRNA on the opposite site of LSU rRNA domain III relative to rpL2 and rpL43 [2]. The direct contacts between rpL2 and rpL43 in mature LSUs opened up the possibility that their observed stabilized incorporation at the level of 7S pre-rRNA containing LSU precursors might occur in an interdependent way. To test this hypothesis, we analyzed the composition of LSU precursors purified from cells in which expression of rpL2 or rpL43 was shut down.

In addition, conditional expression mutants of RPL25 and RPL21 were included in the analyses. In these mutant strains earlier and later, respectively, nuclear pre-rRNA processing phenotypes were observed when compared to the one seen in mutants of RPL2 or RPL43 [65]. In the absence of expression of rpL25 cleavage in the ITS2 region of pre-rRNA separating the 5.8S rRNA from 25S rRNA is very strongly delayed and consequently, production of 7S pre-rRNA and 25.5S pre-rRNA from 27SB pre-rRNA is largely reduced ( [65,70], see scheme in Figure S1). In contrast, in mutants of RPL2, RPL21 and RPL43 7S pre-rRNA is still produced [65]. ^3^H uracil pulse analyses indicated that further conversion of 7S pre-RNA into nuclear forms of processed 5.8S is less tightly blocked after expression shut down of RPL21 compared to phenotypes seen after *in vivo* depletion of rpL2 or rpL43 ( [65], see also scheme in Figure S1).

**Figure 6:**
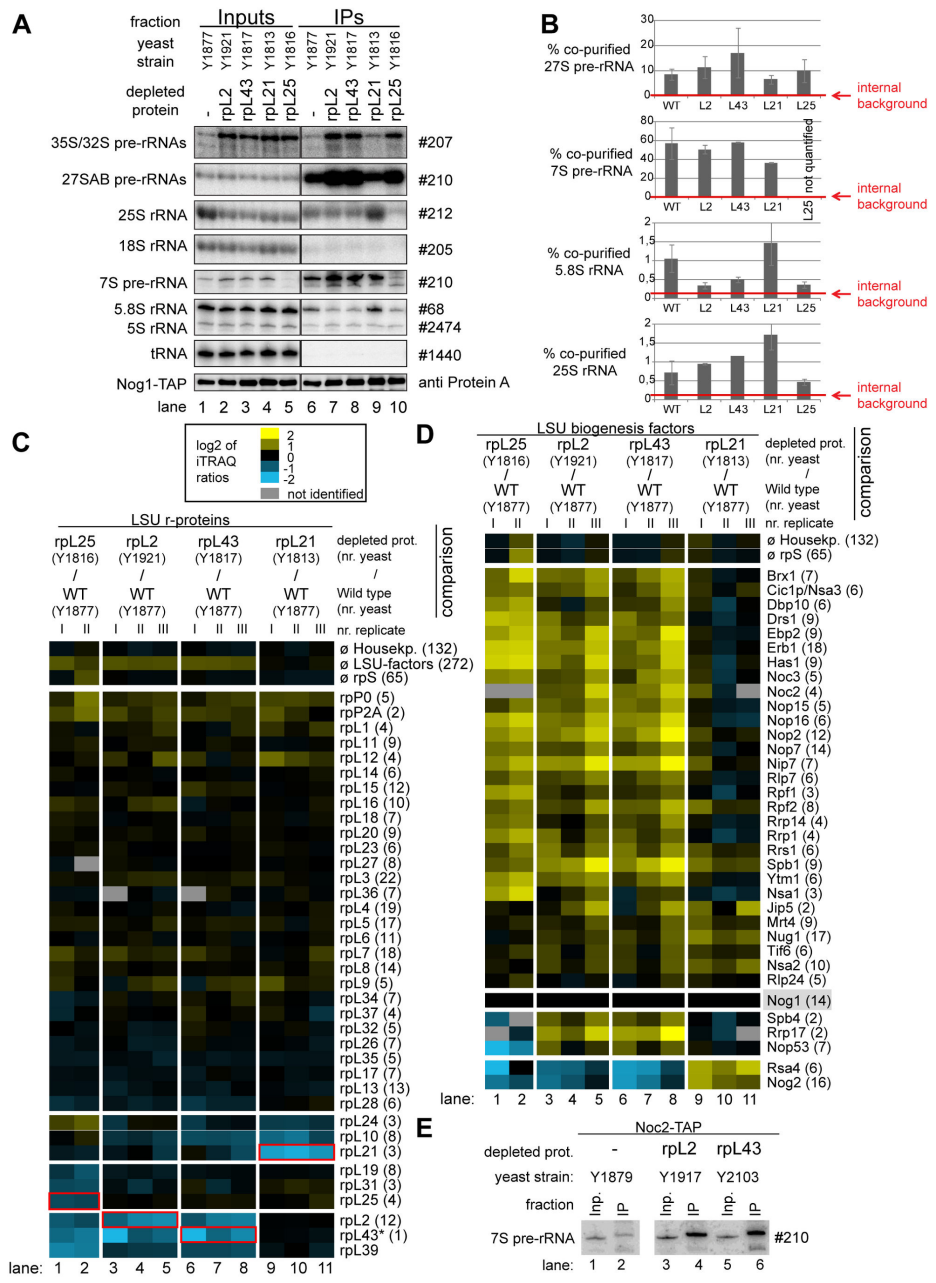
Analyses of the RNA and protein content of pre-60S particles purified via Nog1-TAP after *in* *vivo* depletion of selected ribosomal proteins. The indicated yeast strains expressing a chromosomally-encoded TAP-tagged version of the LSU biogenesis factor Nog1 together with either RPL25, RPL2, RPL43, RPL21 or no LSU r-protein gene under control of the galactose-inducible GAL1/10 promoter were cultivated for four hours in glucose-containing medium to shut down expression of the respective LSU r-protein gene. Nog1-TAP and associated pre-ribosomal particles were then affinity purified from corresponding cellular extracts as described in Materials and Methods. The (pre-) rRNA content of total cellular extracts (Input lanes 1-5) or of parts of the affinity purified fractions (IP lanes 6-10) was analyzed by northern blotting and is shown in (**A**). Detected (pre-) rRNAs are indicated on the left and oligonucleotides used for (pre-) rRNA detection are indicated on the right. Equal signal intensities of the Input and IP fractions correspond to 1% (35S, 27S pre-rRNAs and 25S and 18S rRNAs) or 10% (7S pre-rRNA, 5.8S, 5S rRNAs and glutamyl-tRNA) co-purification efficiencies, respectively. Purification efficiencies of the bait protein Nog1-TAP were monitored by western blotting (see panel designated Nog1-TAP). Equal signal intensities in the western blot analyses indicate 25% purification efficiency of Nog1-TAP. Quantitation of the co-purification efficiencies (in %) of the 7S, 27S, 25S and 5.8S (pre-) rRNAs are shown in (**B**). Red bars in (**B**) designate internal background levels of the affinity purification procedure as measured by co-purification efficiencies of 20S pre-rRNA. One part of the affinity purified fractions was further processed for comparative protein analyses by semi-quantitative mass spectrometry using iTRAQ reagents as described in Materials and Methods. In each experiment particles purified from two strains expressing or not one of the r-proteins of interest were compared. The iTRAQ ratio for each LSU r-protein (**C**) or LSU biogenesis factor (**D**) which was identified by more than one peptide (except rpL43, marked by a (*)) in more than 70% of all comparisons are displayed as a heat map (see color code). A grey color indicates that the respective protein was not identified in the individual experiment shown in this lane. iTRAQ ratios in (**C**) were normalized to the median value of all LSU r-protein ratios. iTRAQ ratios in (**D**) were normalized to the iTRAQ ratio of the bait protein Nog1-TAP. Numbers in brackets behind protein names indicate the average number of peptides by which the respective protein was identified in a single experiment. In (**C**) the iTRAQ ratios of the LSU r-protein whose expression had been shut down are highlighted by red boxes. Several biological replicates of individual experiments are shown in (**C**) and (**D**). (**E**) shows a northern blot analysis of the amount of 7S pre-rRNA co-purifying with Noc2-TAP from extracts of a wild type strain or of conditional expression mutants of RPL2 or RPL43. Cultivation of the strains, affinity purification and northern blotting procedures were done as described above. Same signal intensities detected in the Input and IP fractions correspond to 2.2% purification efficiency of the 7S pre-rRNA.

**Figure 7:**
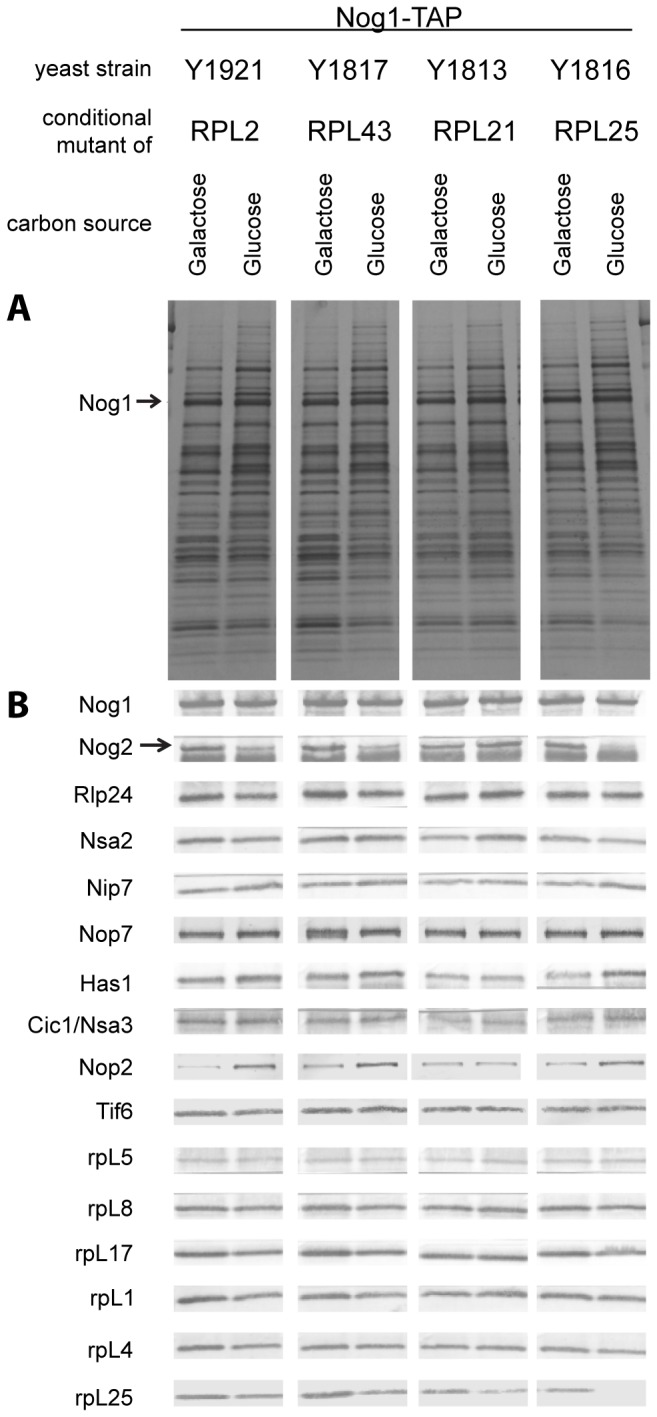
Relative amounts of selected proteins in Nog1-TAP associated LSU precursors depleted of rpL2, rpL43, rpL21 or rpL25 as analyzed by silver staining and western blotting. Nog1-TAP and associated protein were affinity purified from cells of the indicated conditional expression mutants cultivated either in galactose- (left panels) or in glucose-containing (right panels) medium. Affinity purified fractions were analyzed by SDS-PAGE followed by silver staining (**A**) or western blotting (**B**) with appropriate antibodies for the presence of the proteins indicated on the left. The faster migrating of the two bands detected by the anti-Nog2 serum is a cross-contamination with the rabbit IgG.

The respective yeast conditional expression mutants were genetically modified to express a chromosomally encoded TAP-tagged version of the LSU biogenesis factor Nog1 for use in affinity purification of LSU precursors. Nog1 stays associated with intermediate to late LSU precursors [56] and preliminary experiments indicated that its binding to LSU precursors does not require any of the analyzed r-proteins. These conditional mutants and a corresponding wild type strain expressing tagged Nog1 were cultivated for four hours in restrictive conditions (YPD) to shut down (or maintain) expression of individual r-proteins. Total cellular extracts were prepared and pre-ribosomes associated with TAP-tagged Nog1 were affinity purified on an IgG matrix. Results from northern analyses of (pre-) rRNAs contained in the cellular extracts and affinity purified fractions were consistent with the expected pre-rRNA processing phenotypes discussed above (Figure 6A, Input lanes 1-5). In case of the *rpl25* mutant the affinity purified LSU precursor particles contained decreased amounts of 7S pre-rRNA (Figure 6A, lane 10). In case of the *rpl2* and *rpl43* mutants high amounts of 7S- and 27S pre-rRNA were detected (Figure 6A lane 7 and 8) and in case of RPL21, in addition to that, increased amounts of presumably nascent 25S/25.5S and 5.8S/6S rRNA co-purified with Nog1-TAP (Figure 6A, lane 9, see also quantitation in Figure 6B). Part of the affinity purified fractions was used to compare the protein content of particles purified from wild type or mutant cells by semi-quantitative mass spectrometry using iTRAQ reagents (Figure 6C-D). In addition the levels of several LSU r-proteins and biogenesis factors co-purifying with Nog1-TAP were tested by western blotting using specific antibodies (Figure 7). These analyses indicated that for all analyzed strains, a large group of LSU r-proteins co-purified at least equally well with LSU precursors purified from wild type or mutant cells (Figure 6C, r-proteins rpP0 to rpL9; Figure 7). Moreover, increased amounts of a large group of ribosome biogenesis factors including Noc2 were detected in particles purified from mutants of *RPL25*, *RPL43* and *RPL2* (Figure 6D, lanes 1-8, factors Brx1 to Nsa2). In agreement with this, the amount of 7S pre-rRNA associated with Noc2-TAP strongly increased after *in vivo* depletion of rpL2 or rpL43 (Figure 6E, compare 7S pre-rRNA signal in lane 2 with the ones in lanes 4 and 6). We therefore conclude that the release of Noc2 and possibly other factors from LSU precursor particles is affected by lack of assembly of r-proteins as rpL2, rpL43 or rpL25.

A pronounced reduction in the amounts of specific sets of r-proteins and ribosome biogenesis factors was observed in LSU precursors purified from cellular extracts of the different mutant strains. In case of the *rpl25* mutant, characterized by a strong delay in conversion of 27SB pre-rRNA into 7S pre-rRNA ( [65,70], Figure S1), strongest effects were detected on rpL25, rpL31, rpL19 (Figure 6C, lanes 1-2; Figure 7), the rpL2/rpL43/rpL39 group (Figure 6C, lanes 1-2) and on Rsa4, Nog2 and Nop53 (Figure 6D, lanes 1-2; Figure 7). In case of the *rpl2* and *rpl43* mutants, for which previously a delay in processing of 7S LSU pre-rRNA into 5.S rRNA was observed ( [65], Figure S1), the main effects were seen on the rpL2/rpL43/rpL39 group (Figure 6C, lanes 3-8) and on Rsa4 and Nog2 (Figure 6D, lanes 3-8; Figure 7). Analyses of the association of ectopically encoded tagged rpL2 with LSU precursors containing 7S pre-rRNA in cells either expressing or not rpL43 further confirmed interdependent establishment of robust assembly states of rpL2 and rpL43 (Figure S6).

In the *rpl21* mutant strain, none of the r-proteins or factors mentioned above, except for rpL21, was strongly reduced in LSU precursors affinity purified via Nog1-TAP (Figure 6C and 6D, lanes 9-11; Figure 7). In contrast, the levels of Rsa4 and Nog2 even increased after *in vivo* depletion of rpL21 (Figure 6D, lanes 9-11, Rsa4p and Nog2p and Figure 7) indicating a delay in the release of these factors from accumulating LSU precursors. Mild effects on rpL10 and rpL24, members of the group of putatively late assembling r-proteins (group 1B in Figure 4), were observed (Figure 6C). Other members of the group of putatively late assembling r-proteins (rpL29, rpL40 and rpL42) were not detected in these experiments.

### Impact of rpL21 on the release of LSU biogenesis factors from late nuclear LSU precursor particles

**Figure 8:**
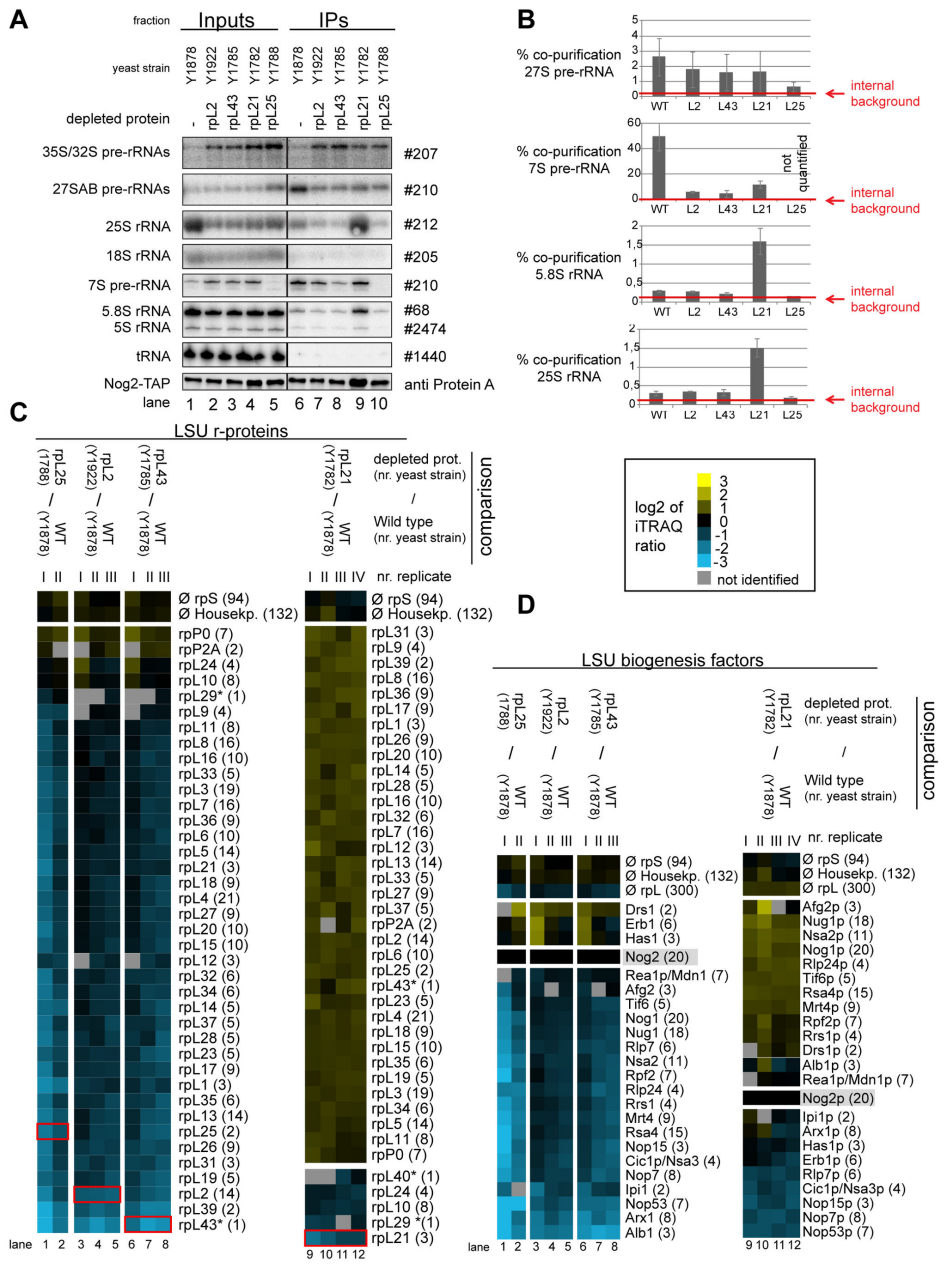
Analyses of the RNA and protein content of pre-60S particles purified via Nog2-TAP after *in* *vivo* depletion of selected ribosomal proteins. Yeast strains expressing a chromosomally encoded TAP-tagged version of the LSU biogenesis factor Nog2 together with either RPL25, RPL2, RPL43, RPL21 or no LSU r-protein gene under control of the galactose-inducible GAL1/10 promoter were cultivated for four hours in glucose containing medium to shut down expression of the respective LSU r-protein gene. Nog2-TAP and associated pre-ribosomal particles were then affinity purified from corresponding cellular extracts as described in Materials and Methods. The (pre-) rRNA content of total cellular extracts (Input lanes 1-5) or of the affinity purified fractions (IP lanes 6-10) are shown in (**A**) as analyzed by northern blotting (see Materials and Methods). Detected (pre-) rRNAs are indicated on the left and oligonucleotides used for (pre-) rRNA detection are indicated on the right. Equal signal intensities of the Input and IP fractions correspond to 1% (35S, 27S pre-rRNAs and 25S and 18S rRNAs) or 10% (7S pre-rRNA, 5.8S, 5S rRNAs and glutamyl-tRNA) co-purification efficiencies, respectively. Purification efficiencies of the bait protein Nog2-TAP were monitored by western blotting (see panel designated Nog2-TAP). Equal signal intensities in the western blot analyses indicate 25% purification efficiency of Nog2-TAP. Quantitation of the co-purification efficiencies (in %) of the 7S, 27S, 25S and 5.8S (pre-) rRNAs are shown in (**B**). Red bars in (**B**) designate internal background levels of the affinity purification procedure as measured by co-purification efficiencies of 20S pre-rRNA. One part of the affinity purified fractions was further processed for comparative protein analyses by semi-quantitative mass spectrometry using iTRAQ reagents as described in Materials and Methods. In each experiment particles purified from two strains expressing or not one of the r-proteins of interest were compared. The iTRAQ ratio for each LSU r-protein (**C**) or LSU biogenesis factor (**D**) which was identified by more than one peptide (except rpL43, rpL29 and rpL40, marked by a (*)) in more than 70% of all comparisons are displayed as a heat map (see color code). A grey color indicates that the respective protein was not identified in the individual experiment shown in this lane. iTRAQ ratios in (**C**) and (**D**) were normalized to the iTRAQ ratio of the bait protein Nog2-TAP. Numbers in brackets behind protein names indicate the average number of peptides by which the respective protein was identified in a single experiment. In (**C**) the iTRAQ ratios of the LSU r-protein whose expression had been shut down are highlighted by red boxes. Several biological replicates of individual experiments are shown in (**C**) and (**D**).

**Figure 9:**
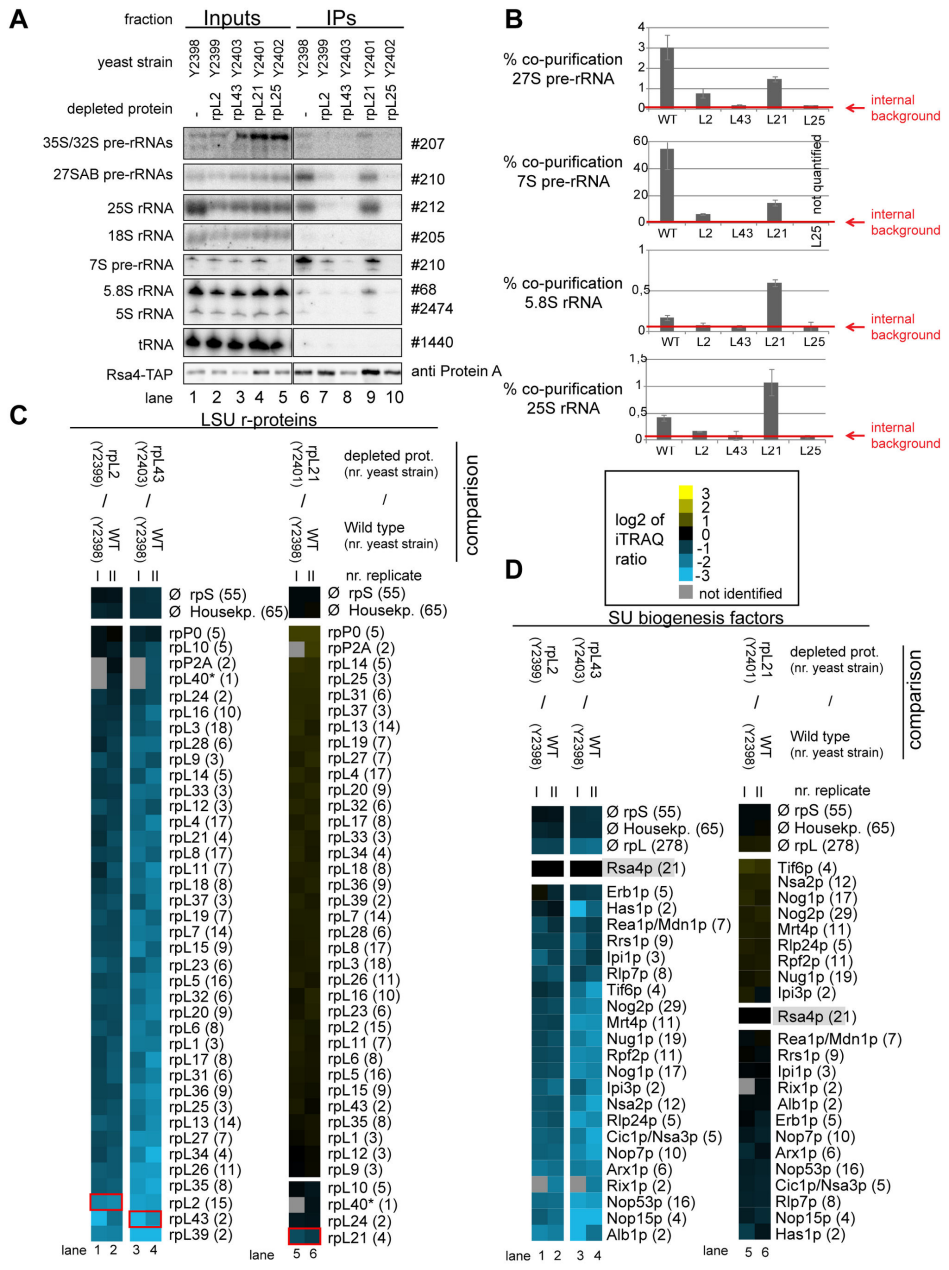
Analyses of the RNA and protein content of pre-60S particles purified via Rsa4-TAP after *in* *vivo* depletion of selected ribosomal proteins. Yeast strains expressing a chromosomally-encoded TAP-tagged versions of the LSU biogenesis factor Rsa4 together with either RPL25, RPL2, RPL43, RPL21 or no LSU r-protein gene under control of the galactose-inducible GAL1/10 promoter were cultivated for four hours in glucose-containing medium to shut down expression of the respective LSU r-protein gene. Rsa4-TAP and associated pre-ribosomal particles were then affinity purified from corresponding cellular extracts as described in Materials and Methods. The (pre-) rRNA content of total cellular extracts (Input lanes 1-5) or of parts of the affinity purified fractions (IP lanes 6-10) are shown in (**A**) as analyzed by total RNA extraction and northern blotting (see Materials and Methods). Detected (pre-) rRNAs are indicated on the left and oligonucleotides used for (pre-) rRNA detection are indicated on the right. Equal signal intensities of the Input and IP fractions correspond to 1% (35S, 27S pre-rRNAs and 25S and 18S rRNAs) or 10% (7S pre-rRNA, 5.8S, 5S rRNAs and glutamyl-tRNA) co-purification efficiences, respectively. Purification efficiencies of the bait protein Rsa4-TAP were monitored by western blotting (see panel designated Rsa4-TAP). Equal signal intensities in the western blot analyses indicate 25% purification efficiency of Rsa4-TAP. Quantitation of the co-purification efficiencies (in %) of the 7S, 27S, 25S and 5.8S (pre-) rRNAs are shown in (**B**). Red bars in (**B**) designate internal background levels of the affinity purification procedure as measured by co-purification efficiencies of 20S pre-rRNA. One part of the affinity purified fractions was further processed for comparative protein analyses by semi-quantitative mass spectrometry using iTRAQ reagents as described in Materials and Methods. In each experiment particles purified from two strains expressing or not one of the r-proteins of interest were compared. The iTRAQ ratio for each LSU r-protein (**C**) or LSU biogenesis factor (**D**) that was identified with more than one peptide (except rpL40, marked by a (*)) in more than 70% of all comparisons are displayed as a heat map (see color code). A grey color indicates that the respective protein was not identified in the individual experiment shown in this lane. iTRAQ ratios in (**C**) and (**D**) were normalized to the iTRAQ ratio of the bait protein Rsa4-TAP. Numbers in brackets behind protein names indicate the average number of peptides by which the respective protein was identified in a single experiment. In (**C**) the iTRAQ ratios of the LSU r-protein whose expression had been shut down are highlighted by red boxes. Several biological replicates of individual experiments are shown in (**C**) and (**D**).

To further characterize the differential impact of rpL2, rpL43, rpL21 and rpL25 on LSU precursor protein composition, the respective conditional expression mutants of these r-proteins together with a corresponding wild type strain were genetically modified to express chromosomally encoded TAP tagged versions of either Rsa4 or Nog2. The various r-protein genes were either expressed or repressed for four hours and Nog2-TAP and Rsa4-TAP were affinity purified from total cellular extracts. Northern blot analyses confirmed that, as shown in Figure 2, Nog2 and Rsa4 associated in wild type conditions with some LSU precursors containing 27SB pre-rRNA (2-3% purification efficiency), with significant populations of LSU precursors containing 7S pre-rRNA (>20% purification efficiency, which is in the range of bait protein purification efficiency) and possibly with some amounts of nascent LSUs containing further processed 25.5S/25S and 6S/5.8S pre-rRNAs (Figure 8A-B and Figure 9A-B). After *in vivo* depletion of rpL2, rpL43 and rpL25 the association of Nog2 with LSU precursors containing 27S and 7S pre-rRNA was clearly reduced, but not completely abolished (Figure 8A-B). The loss of robust association of Rsa4 with pre-ribosomes containing 7S and 27S pre-rRNAs was evident in all the three mutants (Figure 9A-B).

Consistent with the analyses shown in Figure 6D, both Nog2 and Rsa4 associated with increased amounts of LSU precursors containing 25.5S/25S and 5.8S/6S rRNA after expression shut down of rpL21 (Figure 8A-B and Figure 9A-B, lane 9). Altogether, these results further suggested that Nog2 and Rsa4 stay stably associated with nuclear LSU precursors depleted of rpL21. One part of the affinity purified fractions was again used to compare the protein content of particles purified from extracts of mutant cells with the one of particles purified from extracts of wild type cells. From the previous results it was expected that the amounts of most r-proteins and ribosome biogenesis factors co-purifying with Rsa4 and Nog2 decreased due to the presumably weakened association of Rsa4 and Nog2 with LSU precursors in mutants of *RPL2*, *RPL43*, and *RPL25*. Indeed, the observed results were in line with these expectations (Figure 8C lanes 1-8 and Figure 9C, lanes 1-4). In agreement with the previous RNA analyses (Figures 8A and 9A), *in vivo* depletion of rpL21 did not substantially affect the association of Nog2 or Rsa4 with LSU precursors as indicated by an efficient co-purification of most LSU r-proteins and biogenesis factors (log_2_ ratios > =0 in Figure 8C, lanes 9-12 and 9C, lanes 5-6). As seen before with particles purified via tagged Nog1, the main effects of rpL21 depletion on r-protein composition were seen on rpL21 itself, and to a clearly weaker degree on rpL10, rpL24, rpL40 and, when detected, rpL29 (Figures 8C and 9C lanes 9-12 and 5-6, respectively). Some of the early recruited biogenesis factors as Nop7, Erb1, Nsa3 and Rlp7 were underrepresented in particles purified via Nog2-TAP from extracts of the RPL21 mutant (Figure 8D, lanes 9-12). The same was true for Nop53 which preferentially binds to intermediate and late nuclear LSU precursors ( [75], see also Figure 2). These effects were less evident in rpL21 depleted LSU precursors co-purifying with Nog1 or Rsa4 (Figure 6D, lanes 9-11 and Figure 9D, lanes 5-6, respectively). These observations suggested that Nog2 preferentially associates with specific sub-populations of rpL21 depleted LSU precursor particles which partially released factors as Nop7, Erb1, Rlp7, Nsa3 and Nop53. Indeed, a direct comparison of the (pre)-rRNA content found in the rpL21 depleted purificates of the different bait proteins Nog2-TAP, Rsa4-TAP and Nog1-TAP indicated that the Nog2-TAP associated LSU precursor populations were characterized by very high ratios of late nascent (pre-) rRNAs (25.5/25S and 6S/5.8S pre-rRNAs) versus intermediate pre-rRNAs (27S pre-rRNA and 7S pre-rRNA) (see Supplementary Figure S7A and quantitation in Figure S7C). To further analyze a possible release of specific ribosome biogenesis factors from Nog2 associated late nuclear LSU precursors depleted of rpL21, yeast expression mutants of RPL21 were created which encoded tagged versions of either Nop7 or Nop53. Tagged Nop53 and Nop7 co-purified 27SB and 7S pre-rRNA from cellular extracts of a RPL21 mutant strain with similar efficiencies as seen in a wild type situation (Figure S7B and C). But, in sharp contrast to what was observed before for Nog2-TAP (Figure 8A-B), association of Nop53-TAP or Nop7-TAP with nascent LSU precursors containing 6S/5.8S (pre-) rRNA did not increase over background after *in vivo* depletion of rpL21(Figure S7B-C). In sum, these observations provided further evidence that a specific group of LSU biogenesis factors including Nop53 and Nop7, and possibly Erb1, Rlp7, Nop15 and Nsa3 (Figure 8D), partially dissociates from the largely processed nuclear LSU precursor population made in the absence of rpL21. On the other hand, the release of a few other factors, including Nog2, Rsa4, Rrs1 and Rpf2 (Figure 8D) seemed to be inefficient.

## Discussion

### General and specific assembly characteristics of yeast LSU r-proteins

The results shown here suggest that a rather loose association of many yeast LSU r-proteins with early LSU precursors is progressively converted into a more robust interaction during particle maturation. Indications for a similar, progressive assembly behavior of single yeast SSU r-proteins came from a previous report analyzing the association of tagged SSU r-proteins with SSU precursors of different maturation states [71]. Moreover, studies using a factor-free, prokaryotic SSU *in vitro* assembly system suggested that weak initial interactions of most r-proteins with SSU rRNA are progressively stabilized during the assembly process [15,16,94]. Conversion of loose into tight association with LSU rRNA was also observed for a subset of LSU r-proteins in a defined two-step *in vitro* reconstitution system of prokaryotic LSUs [95]. Therefore, the establishment of a robust interaction of many r-proteins with ribosomal subunits seems to occur in a multistep fashion, rather than being a one step event.

The experiments shown in Figures 4 and 5 indicate that some LSU r-proteins are characterized by a specific assembly behavior differing from the large majority of yeast LSU r-proteins. One group with a unique assembly behavior consists of rpL2, rpL43 and rpL39. The binding strength of these r-proteins with LSU precursors was observed to increase in a pronounced way concomitant with cleavage in the ITS2 region of LSU pre-rRNA. Interestingly, L2, the only *E. coli* homologue of this group of r-proteins, was found to be under-represented in LSU precursor particles isolated from cellular extracts [96]. *E. coli* L2 was initially classified as primary binder in a defined LSU *in vitro* reconstitution system but more detailed studies indicated that in some circumstances its stable association with 23S rRNA depends on other r-proteins [97,98]. Yeast rpL2, as *E. coli* L2, features distinct interaction interfaces with four of the LSU rRNA secondary structure domains in mature LSUs (LSU rRNA domains II, III, IV and V) [2,9]. We hypothesize that establishment of some of these interactions is sufficient for its detectable, but comparably weak association with early to intermediate nuclear yeast LSU precursors. Interactions of rpL2 with parts of LSU rRNA domain IV, identified in earlier work as being the primary *in vitro* binding site of the *E. coli* homologue of rpL2, probably play a predominant role in this initial association with LSUs [99,100]. The combined interaction with all of the four LSU rRNA secondary structure domains could then lead to its stabilized assembly.

A second group of yeast r-proteins, consisting of the essential r-proteins rpL10, rpL40 and rpL42 and the non essential r-proteins rpL24, rpL29, rpP1A/B and rpP2A/B, was found to be specifically under-represented in most nuclear LSU precursor populations. In agreement with this, previous studies in yeast and/or mammals suggested late assembly for rpL10, rpL24, rpL29, rpL40 and rpP2 in eukaryotes. *E. coli* L16, which shares homology with yeast rpL10, was found only in trace amounts in the first intermediates of *in vitro* reconstituted prokaryotic LSUs [95]. Both L16 and the rpP1 homologue L12 were classified as being under-represented in LSU precursor particles isolated from cellular extracts of *E. coli* [96,101]. Except phospho-stalk proteins and rpL24, all other r-proteins in this group (rpL10, rpL29, rpL40, rpL42) bind in and around the central protuberance which is made of 5S rRNA, parts of LSU rRNA domains II and V and several r-proteins [2]. Accordingly, 5S rRNA and some directly interacting r-proteins are recruited rather early during yeast ribosome maturation [31] but the exact orientation of the central protuberance in the LSU seems to involve several late r-protein assembly events. Remarkably, despite the divergence in the composition of prokaryotic and eukaryotic ribosomes and the factors involved in their maturation, very similar conclusions were drawn in a recent study analyzing assembly states of r-proteins in LSU precursors and mature LSUs isolated from *E. coli* [101]. The proposed exchangeability of eukaryotic rpL10 and phospho-stalk proteins rpP1/rpP2 furthermore suggests that the structural organization of the phospho-stalk and the central protuberance is subject to dynamic changes even in mature LSUs. In line with this, recent single-molecule cryo-electron microscopic analyses indicated that in LSU particles associated with Arx1, which binds to late LSUs precursors and free mature LSUs (Figure 2 and [61,78–80]), the definite organization of the central protuberance and the phospho-stalk, including correct assembly of rpL10 and rpP1/rpP2 proteins, is not accomplished [55].

### Hierarchical interrelationships between selected assembly events in yeast – a possible impact of direct interactions between r-proteins and the folding state of rRNA secondary structure domains

To initially address how some of the LSU r-protein-rRNA assembly events are controlled *in vivo*, we analyzed the changes in pre-ribosomal composition in conditional expression mutants of some yeast r-protein genes. In general, shutting down the expression of the vast majority of essential ribosomal protein genes in yeast leads to some destabilization and turnover of deficient ribosomal precursor particles. Consequently, only changes in the composition of LSU particles not (yet) degraded, could be analyzed in this experimental setup. We focused on analyzing in more detail the observed stabilized incorporation of the rpL2 group of r-proteins which correlated with nuclear endonucleolytic cleavage at C_2_ site in the ITS2 of LSU pre-rRNA. Expression shut down of rpL25 leads to a delay of this cleavage event [65,70]. According to the results shown in Figure 6, *in vivo* depletion of rpL25 affected predominantly the stabilized incorporation of rpL2, rpL43, rpL31, rpL19 and rpL39 into LSU precursors. The degree of the observed effects did not argue for the affected r-proteins being completely removed from the analyzed assembly intermediates but rather that a specific step in the progressive assembly process of the respective r-proteins was disturbed. Remarkably, in the structure of mature LSUs [2], rpL25, rpL2, rpL43, rpL31, rpL19 and rpL39 show interactions with secondary structure domain III of LSU rRNA which occupies a defined space next to the 5.8S rRNA. The primary *in vitro* binding site of yeast rpL25 and of its *E. coli* homologue L23, was shown to be in LSU rRNA domain III [99,100,102]. Accordingly, yeast rpL25 might be required for proper folding of LSU rRNA domain III to enable stabilized association of rpL2, rpL43, rpL31, rpL19 and rpL39 with LSU precursors. Alternatively, blocking rpL25 dependent rRNA cleavage at site C_2_ might interfere with stabilized assembly of these rRNA domain III binders. Future analyses on the LSU precursor composition after expression shut down of other r-proteins which are required for C_2_ cleavage but which show no binding sites in rRNA domain III in the LSU might help to distinguish between these possibilities. These analyses should also shed more light on what the redundant effects of rpL25 and other r-proteins required for C_2_ cleavage are on the LSU precursor association of ribosome biogenesis factors, which might play specific roles in stabilized association of rpL2, rpL43, rpL31, rpL19 and rpL39.

After *in vivo* depletion of either rpL2 or rpL43 the cleavage at site C_2_ in the ITS2 of LSU pre-rRNA still takes place, but downstream trimming of the ITS2 pre-rRNA is significantly delayed [65]. In this situation specific effects on levels of rpL2, rpL43 and rpL39, but not on levels of rpL25, rpL19 and rpL31 in LSU precursor particles were detected. Accordingly, cleavage at site C2 in the ITS2, even if possibly required (see discussion above), seems not to be sufficient for stabilized incorporation of rpL2, rpL43 and rpL39 into LSU precursors. The results indicate furthermore that establishment of robust assembly states of rpL2 and rpL43 is interdependent but not required for assembly of rpL25. This interdependence could in part be explained by the direct interactions between rpL2 and rpL43 seen in mature LSUs [2]. Together with the previous results these data argue for a hierarchy of assembly events of some LSU rRNA domain III binding r-proteins which is linked to processing of ITS2 pre-rRNA spacer sequences.

Yeast rpL21 localizes in mature ribosomes in the central protuberance near the 5S RNP, and therefore far from LSU rRNA domain III [2]. In conditional expression mutants of rpL21, late nuclear ITS2 pre-rRNA processing proceeds at substantially higher rates than in mutants of rpL2 or rpL43 [65]. On the other hand, efficient nuclear export of the LSU particles could not be detected. Analysis of LSU precursor particle compositions now indicated that assembly of none of the LSU rRNA domain III binders discussed above was significantly affected after expression shut down of rpL21. In contrast, some effects were seen on the group of late assembling r-proteins rpL24, rpL29, rpL40 and rpL10, even if the bait proteins chosen in these experiments (Nog1, Nog2 and Rsa4) co-purified, in wild type conditions, populations of LSU precursors in which these proteins were underrepresented (Figure 4, lanes 14-17 and 22-23). Except for rpL24, binding sites of these proteins in mature ribosomes cluster around rpL21 in the central protuberance, and rpL21 directly contacts rpL10 and rpL29 in mature LSUs. The effects detected here in the absence of rpL21 on stabilized incorporation of rpL29, rpL40, rpL10 might therefore occur due to changes in folding states of rRNA domains II and V around the central protuberance. Alternatively, assembly of these proteins, and rpL24, might require nuclear export of LSU precursors which we failed to detect in the conditional expression mutant of rpL21.

The ensemble of data collected up to now might give first insights on general patterns which emerge for interrelationships between yeast r-protein assembly events *in vivo*. A common theme seems that specific assembly interrelationships can be detected between directly interacting proteins and binders of the same rRNA secondary structure domain. Examples for this are the SSU head domain binders rpS5 and rpS15 which were suggested to affect several r-protein assembly events in the head domain [71]. Similarly, previous work established assembly interrelationships between the two yeast 5S rRNA interacting proteins rpL5 and rpL11 [31]. A detailed comparison of LSU precursor particles depleted of either rpL7 or rpL8 indicated that, although they bind to rather distinct places in mature LSUs, both affect assembly of a common group of r-proteins. However, it became evident that each has in addition to that a specific and pronounced impact on assembly events involving a set of surrounding proteins in either LSU rRNA domain II or domain I, respectively [29]. Proteins whose assembly was specifically affected in the RPL7 mutant strain (rpL6, rpL33, rpL7, rpL20, rpL14) or the RPL8 mutant strain (rpL8, rpL13, rpL15, rpL36) form in mature LSUs two networks of local protein–protein interactions involving rpL7 or rpL8, respectively. In the present work, a hierarchy of pronounced local assembly effects was detected among members of a group of r-proteins (rpL25, rpL43, rpL2, rpL19, rpL31, rpL39) interacting with LSU rRNA domain III. Among them, interdependent stabilized incorporation into LSU precursors was observed for rpL2 and rpL43 which directly interact with each other in mature ribosomes. In addition to that, rpL21 was seen to be required for stabilized assembly of a few r-proteins which participate in anchoring 5S rRNA in the LSU central protuberance through an interaction network involving LSU rRNA domains II and V. Assembly interrelationships between directly interacting r-proteins and binders of the same rRNA secondary structure domain were also frequently detected in the *in vitro* reconstitution systems of prokaryotic ribosomal subunits (see 100,103). In fact, most of the *in vitro* assembly interdependencies observed for the SSU were restricted to r-proteins binding in the same secondary structure domain (reviewed in 12). More recent data indicated that in case of bacterial L5 (homologue of yeast rpL11), L22 (homologue of yeast rpL17) and L15 (homologue of yeast rpL28), effects on assembly of other r-proteins *in vivo* are restricted to direct interaction partners and their local surroundings in LSUs [104,105]. Altogether, these studies suggest that the hierarchy of r-protein assembly events is in part determined by direct interactions between r-proteins and by effects on folding in individual rRNA secondary structure domains, *in vitro* and *in vivo*.

### Similarities and differences in the hierarchy of r-protein assembly events *in vivo* and *in vitro*

The results of these and previous studies [29,31,71] suggest that yeast r-protein assembly events are *in vivo* in part governed by hierarchical and cooperative relationships among each other. These interrelationships might directly relate to the factor independent assembly hierarchy seen in prokaryotic ribosome *in vitro* assembly systems. Previously, evidence was shown for a partial overlap between the hierarchy of specific assembly events in the yeast SSU head domain and the well characterized factor independent *in vitro* assembly hierarchy of prokaryotic SSU r-proteins [71]. On the other hand, no clear evidence could be observed for several interrelationships between yeast r-protein assembly events predicted from the prokaryotic *in vitro* assembly maps. These include the lack of detected effects of yeast rpS5 (bacterial S7) depletion on assembly of rpS0 (bacterial S2) [71] and of rpL26 (bacterial L24) depletion on assembly of rpL17 (bacterial L22) and rpL35 (bacterial L29) [59]. In the present work, only very mild effects were observed for rpL2 (bacterial L2) depletion on assembly of rpL28 (bacterial L15), and for rpL25 (bacterial L23) depletion on assembly of rpL35 (bacterial L29). No effect was detected for rpL2 (bacterial L2) depletion on assembly of rpL11 (bacterial L5). The effects observed here on stable assembly of rpL2 (bacterial L2) by *in vivo* depletion of rpL25 (bacterial L23) and the effects on assembly of rpL17 (bacterial L22) and rpL26 (bacterial L24) by *in vivo* depletion of rpL7 (bacterial L30) [29] were not predicted from the *in vitro* assembly maps. It should be noted again that most yeast r-proteins seem to counteract degradation of (pre-)ribosomal particles (for an example, see 65) and it can therefore not be excluded that certain populations of misassembled particles made in r-protein gene mutants in yeast cells are turned over faster than others. Such selective degradation of particles of different assembly states, which is unlikely to occur in the *in vitro* reconstituted ribosome assembly systems, could have clearly affected the outcome of these studies. It can also not be excluded that some of the assembly effects seen here *in vivo* are due to expression feedback regulation among r-protein genes (see 106 for a discussion on this topic). In addition, quite a number of r-proteins are exclusively found either in bacteria or in eukaryotes [1], and together with species-specific variations in the primary structure of r-proteins and rRNA, these aspects could lead to differences in the intrinsic hierarchy of individual assembly events. Apart from this, yeast mutant studies resemble most likely in many cases “single omission” experiments while the assembly interrelationships in the LSU *in vitro* assembly maps were deduced from experiments involving a defined subset of r-proteins and rRNAs (see as example [107]). This reductionist approach could have led to the detection of assembly interrelationship not seen *in vivo*. That might be especially relevant in the case of r-proteins which establish interactions with several domains of rRNA, a feature seen for many LSU r-proteins. A good example for this is *E. coli* L5 (yeast rpL11) whose interaction with 23S rRNA was seen to depend *in vitro* on the presence of L2 (yeast rpL2) in an experimental setup including only L2, L5 and 23S rRNA [107]. Both *E. coli* L5 and yeast rpL11 interact directly with LSU rRNA domain V and with 5S rRNA, which itself has a number of contact points within the LSU [2,9]. Therefore, the observation made here that the yeast L5 homologue rpL11 can still be efficiently recruited to LSU precursors in the absence of rpL2 expression is likely due to the L2 independent association of the rpL11-5S RNP with ribosomes. The different set of players present in *in vitro* assembly experiments or in cells of r-protein gene mutants could also explain why some bacterial genes coding for r-proteins with defined roles in the *in vitro* ribosomal subunit assembly hierarchy were found to be not essential for cellular growth (reviewed in 108] [105,109, and references therein). In addition, as shown for *E. coli* L24 (homologue of yeast rpL26) and L15 (homologue of yeast rpL28), both encoded by non-essential genes, the importance of r-proteins for LSU assembly *in vitro* can substantially depend on the exact reconstitution conditions, as the concentration of salt and the temperature chosen for specific steps of the assembly reaction [110,111]. Future work in *vitro* and *in vivo* will have to delineate in more detail how other aspects of ribosome assembly *in vivo* which were not reproduced in the original prokaryotic *in vitro* reconstitution systems influence the kinetics and the hierarchy of r-protein assembly events. Among them are the presence of specific ribosome biogenesis factors, different pre-rRNA processing and modification states and the initiation of assembly events already during ongoing synthesis of rRNA.

### Interplay between r-protein assembly events and the transient association of ribosome biogenesis factors with precursor LSUs

It was previously shown that at least some of the eukaryotic ribosome biogenesis factors have impact on the assembly states of r-proteins [25,31–33] and that individual r-proteins can influence the recruitment or release of biogenesis factors [29–31,33–35]. In this work we provide evidence that the LSU rRNA domain III binder rpL25 is required for efficient recruitment of three specific LSU biogenesis factors, Rsa4, Nog2 and Nop53 to LSU precursors. Association of Rsa4 and Nog2, not of Nop53, with LSU precursors was also affected after expression shut down of rpL2 or rpL43, whose stable assembly depends on rpL25. Moreover, the experiments pointed towards a delay of the release of a group of early acting ribosome biogenesis factors, including Noc2, from LSU precursors in mutants of RPL2, RPL25 and RPL43. Interestingly, the pre-rRNA processing phenotypes previously observed for mutants of Nop53, Nog2, and Rsa4 strongly resemble the ones seen after expression shut down of rpL2 or rpL43 [53,75,81–83]. In all cases 27SB pre-rRNAs, but also 7S pre-rRNAs accumulate (Figure S1). Accordingly, the pre-rRNA processing phenotype seen in mutants of rpL2 and rpL43 might be due to inefficient recruitment of Nog2 and Rsa4 to LSU precursors. The endonucleolytic cleavage at site C_2_ in the ITS2 spacer seems less affected in all these cases than in mutants of rpL25 for which only accumulation of 27SB pre-rRNA, not of the cleavage product 7S rRNA, was detected. Therefore, inefficient cleavage at C_2_ seen after expression shut down of rpL25 cannot be easily explained by reduced LSU precursor association of Nog2, Rsa4 or Nop53, which seem to be primarily required for downstream events in LSU pre-rRNA processing. Consequently, interactions of rpL25 with LSU rRNA might have a more direct role in cleavage at site C_2_ of LSU pre-rRNA.

Further analyses suggested that *in vivo* depletion of rpL21 primarily affected the ribosome biogenesis factor composition of late nuclear LSU precursor particles. A late LSU precursor population accumulated in the rpL21 expression mutant which contained largely processed rRNAs. In wild type cells, pre-ribosomes containing these rRNAs are exported to the cytoplasm [92] but in case of rpL21 mutants they seem to be retained in the nucleus [65]. This late population of nuclear entrapped LSU precursors could be rather specifically enriched from yeast extracts by affinity purification of tagged Nog2, therefore allowing a more detailed analysis of its composition (Figure 8D). Evidence was seen for a (partial) release of some ribosome biogenesis factors from these late nuclear-entrapped LSU precursors. Among them were Erb1 and Nop7 whose removal from LSU precursors was previously shown to depend on the ATPase Rea1 [112]. Interestingly, other LSU biogenesis factors were still well-represented in late nuclear LSU particles accumulating after *in vivo* depletion of rpL21. Some of these factors, such as Tif6 and Rlp24, are thought to accompany LSU precursors to the cytoplasm where they are finally released (reviewed in 36). In contrast, another group of these factors, including Nog2, Rsa4, Ipi1, Rea1, Rpf2 and Rrs1, are most likely removed from LSU precursors prior to nuclear export (reviewed in 21). As for Nop7 and Erb1, there is good evidence that the efficient release of Ipi1 and Rsa4 from nuclear LSU precursors involves the ATPase activity of Rea1 [76]. Therefore the data suggest that while assembly of rpL21 is not strictly required for a first round of Rea1 dependent release of early acting ribosome biogenesis factors as Erb1 and Nop7 from LSU precursors, it is a prerequisite for efficient Rea1p mediated removal of later acting factors as Rsa4 and Ipi1. Moreover, rpL21 seems to be involved in the efficient dissociation of Rpf2 and Rrs1 from late nuclear LSU precursors. These two proteins were previously shown to facilitate the incorporation of 5S rRNA together with rpL5 and rpL11 into pre-ribosomes [31]. Interestingly, rpL21 binds in mature ribosomes to LSU rRNA domains II and V at the base of the central protuberance which is mainly made of the 5S RNP. The release of Rpf2 and Rrs1 from LSU precursors seems thus to involve local changes in the assembly and folding states of LSU rRNA domains II and V.

Certainly, the determination of pre-ribosomal compositions in mutants of other LSU r-proteins should allow one to draw further conclusions on the interplay of r-protein assembly events, the dynamic association of ribosome biogenesis factors with precursors LSUs, and processing and transport of pre-rRNAs. 

## Materials and Methods

### Yeast strains & microbiological procedures

Yeast strains expressing chromosomally-encoded TAP-tagged LSU biogenesis factors (Noc2, Nog1, Nog2, Rsa4, Nop53, Nop7, Arx1) were created according to [113]. Primers used for amplification of the TAP-URA3-cassette from plasmid pBS1539 are listed in Figures S2 and S4. The resulting PCR product was transformed into yeast cells [114] and the correct genomic integration of the TAP-URA3 cassette was verified by selection for uracil prototrophy on appropriate minimal medium (SGC-URA), followed by western blotting analyses for detection of the respective TAP-fusion proteins. The yeast strains expressing FLAG or GFP tagged LSU r-proteins were created as follows: a plasmid coding for the tagged LSU r-protein under the control of a constitutive promoter (pRPS28Bprom-TAG-RPLX; URA3 or pRPS28Bprom-RPLX-TAG; URA3; see Figure S3 for details) was transformed into the respective LSU r-protein gene(s) deletion strain carrying a plasmid (pGAL-RPLX; LEU2; CEN, see Figure S2 for details) complementing the lethal phenotype of the LSU r-protein gene deletions. After transformation and selection for uracil prototrophy (SGC-URA plates), the strains were transferred to glucose containing medium. All plasmids coding for tagged r-proteins used in this study complemented the expression shut down of the corresponding essential r-protein genes. Generation times of yeast strains expressing FLAG-fusion alleles of r-proteins were determined as described in [115]. Significant reduction in generation times were reproducibly observed for strains Y485 and Y1212 expressing Flag-fusion alleles of RPS26 and RPL40, respectively (see generation times in Figure S2). Some minor growth phenotype was observed for yeast strains expressing carboxy-terminal TAP fusion proteins of Nog1p. A detailed description of yeast strains, plasmids, and oligos used in this study can be found in Figure S2, S3 and S4, respectively. Yeast strains conditionally expressing LSU r-protein genes were cultivated at 30°C in YPG (1% yeast extract, 2% bacto peptone, 2% galactose); expression of the respective genes was shut down by incubating cells in YPD (1% yeast extract, 2% bacto peptone, 2% glucose) for 4 hours at 30°C.

### Affinity purification of (pre-) rRNPs using IgG coupled magnetic beads followed by semiquantitative mass spectrometry

TAP-tagged LSU biogenesis factors and associated preribosomal particles were purified from total cellular extracts in one step using rabbit IgG coupled to magnetic beads as described in [33,116] with minor modifications. A cell pellet corresponding to 1 l yeast culture with OD600 = 0.8-1.2 was resuspended in 1.5 ml of cold MB buffer (20 mM Tris HCl pH 8, either 200 mM or 300mM KCl, 5 mM MgOAc, 2 mM Benzamidine, 1 mM PMSF, and 0.04 U/mL RNasin) per g of cell pellet; 0.8 mL of this cell suspension was added to 1.4 ml glass beads (Ø 0.75–1 mm) and divided into 2-ml reaction tubes. A cell lysate was prepared by vigorous shaking of the cell suspension in an IKA-Vibrax VXR shaker at 4°C for 15 min, followed by 2 min on ice. This procedure was repeated twice. The cell lysate was cleared from cell debris by two centrifugation steps, once for 5 minutes at 14,000 rounds per minute and once for 10 minutes at 14,000 rounds per minute in a table top centrifuge at 4°C. The protein concentration of the cleared lysate was determined using the Bradford assay. Triton X-100 and Tween 20 were added to the cell lysate to final concentrations of 0.5% (w/v) and 0.1% (w/v), respectively. One percent (v/v) of the lysate were taken for Western and Northern blotting analyses, respectively (“Input” samples). Equal amounts of cell lysate (typically 1 ml with 20–50 mg of total protein) were incubated for 1 h at 4°C with 200 µl of an IgG (rabbit serum, I5006-100MG, Sigma)-coupled magnetic beads slurry (1 mm BcMag, FC-102, Bioclone) equilibrated in MB buffer containing 0.5% Triton X-100 and 0.1% Tween. The beads were washed four times with 1 ml cold MB buffer with 0.5% Triton X-100 and 0.1% Tween 20. Twenty percent of the suspension was taken for RNA analyses by Northern blotting. The remaining part of the suspension was washed two times with 1 ml AC buffer (100 mM NH_4_OAc pH 7.4, 0.1 mM MgCl_2_) to remove remaining salt from the sample. Bound proteins were eluted two times with 500 µl of freshly prepared 500 mM NH_4_OH solution for 20 min at RT. Both eluate fractions were pooled, lyophilized overnight, and further processed for semi quantitative mass spectrometric protein analyses as described in [33]. Peptides used for protein identification and quantitation were identified with a confidence interval of more than 95%.

### Affinity purification of (pre-) rRNPs using anti-FLAG antibody coupled sepharose beads

FLAG tagged rpS26 (Figures 2-4) or one of 16 FLAG tagged LSU r-proteins (Figures 5 and S5/S6) were purified from total cellular extracts in one step using anti-FLAG coupled sepharose beads. The cellular extracts corresponding to 250 ml yeast culture with OD600 = 0.8-1.2 were produced as described above using the same buffers (containing 200 or 300 mM KCl as indicated in the Figure legends). Equal amounts of cell lysate (typically 0.5 ml with 20–50 mg of total protein) were incubated for 1 h at 4°C with 100 µl of anti FLAG-coupled M2 beads (Sigma) equilibrated in MB buffer containing 0.5% Triton X-100 and 0.1% Tween. Washing and elution of rpS26-FLAG and associated (pre-) rRNPs for further analyses by semi-quantitative mass spectrometry was performed as described above. Total RNA of the (pre-) rRNPs co-purified via the FLAG-tagged LSU r-proteins was extracted by hot acidic phenol-chloroform treatment (see below).

### Data visualization and hierarchical clustering analyses of semi quantitative proteome data of LSU biogenesis factors and LSU r-proteins present in different (pre-) ribosomal particles

Hierarchical clustering analysis of semi quantitative mass spectrometry data sets derived from several experiments was done as described in [33], using cluster 3.0 software [117]. All observed iTRAQ ratios were expressed in log_2_ scale. To correct for possible experimental bias in the iTRAQ labeling procedure, the median value of the iTRAQ ratios of LSU biogenesis factors or LSU r-proteins was set to zero in each individual experimental data set shown in Figures 3 and 4, respectively. For the analyses of the protein content of pre-60S particles after *in vivo* depletion of LSU r-proteins (shown in Figures 6, 8 and 9) the observed iTRAQ ratios were normalized as indicated in the respective Figure legends. The distance matrices of the data shown in Figures 3 and 4 were calculated by the “City block distance” method and hierarchical clustering analyses were done with the “centroid linkage” algorithm for both comparison of similarities between the different datasets and of the behaviour of proteins in the various experiments. Cluster visualization (tree and heat map) was done with Java Treeview (see http://www.eisenlab.org/eisen/?page_id=42).

### RNA extraction and Northern blotting

RNA was extracted by hot acidic phenol–chloroform treatment as previously described [65]. Northern blotting analyses after RNA separation on formaldehyde/MOPS agarose gel (18S/25S rRNA and their precursors) or Urea/TBE/Polyacrylamid gels (7S pre-rRNA, 5.8S and 5S rRNAs and Glutaryl-tRNA) were done essentially as described in [118]. Hybridization with probes was performed in 50% formamide/5x SSC/0,5% SDS/5x Denhardt’s solution at 30°C with the 32P-labelled DNA oligo nucleotides listed in Figure S4. Northern blots were analyzed using a FLA3000 (FUJI). Data were quantified using the MultiGauge software (FUJI).

### Western Blotting

Levels of TAP-tagged LSU biogenesis factors were determined by Western blotting analysis using PAP visualisation reagent (DakoCytomation, Z 0113) in a dilution of 1:5000. Protein signals were visualised by chemiluminescence using a Fluorescence Image Reader LAS3000 (Fujifilm). Western blotting and immunodetection of ribosome biogenesis factors and r-proteins in affinity purified fractions was performed as described in [32].

### Immuno-electron Microscopy

Yeast cells were cultured in rich YPD medium at 30°C, harvested at exponential phase and fixed for 45 min in 4% paraformaldehyde at room temperature. After washing with 0,1 M Cacodylate buffer, they were incubated with 1% sodium metaperiodate for 1 hour, then treated with 50 mM ammonium chloride for 1 hour. Samples were dehydrated in a graded ethanol series and infiltrated with medium grade L.R. White resin (EMS). The resin was polymerised for 48 h at 50°C. Sections were cut on a Reichert Ultracut microtome. For immunolabeling, polyclonal antibodies raised in rabbit and directed against the GFP were used. Ultrathin sections were mounted on 400 mesh nickel grids. After 15 min on a drop of 10mM Tris buffer (pH 7.6) containing 10% of goat serum, the grids were incubated with the primary antibody (anti GFP 1/50-1/300) diluted in Tris, 1% Bovine Serum Albumine (BSA), 0,1% Tween-20, for 2 hours at room temperature. The sections were washed for 30 minutes with Tris containing 1% BSA. They were then transferred for 1 h to 10 nm colloidal gold-conjugated goat anti-rabbit antibody (BBI) diluted 1/80 in Tris, BSA 1%. After incubation, the grids were washed for 20 minutes with Tris buffer and for 10 min in distilled water. The sections were then air-dried. Controls were performed using gold-labeled antiserum alone. No labeling was detected on these grids. For electron microscopy analysis, sections were contrasted with 5% aqueous uranyl acetate and examined at 80 kV in a Jeol 1200-EX electron microscope.

## Supporting Information

Figure S1 Simplified scheme of post-transcriptional LSU pre-rRNA processing events in the yeast *S. cerevisiae*. Three of the four rRNAs found in the mature ribosome are derived from the polycistronic transcript (**A**) made by RNA polymerase I which is processed through a series of endo- and exonucleolytic reactions. (**A**) illustrates the transcription start site (+1), the external transcribed spacer regions (5’ ETS1, 3’ ETS), the internal transcribed spacer regions (ITS1, ITS2), and the major pre-rRNA processing sites. The sizes of the indicated regions are not in proportion to their real length. The positions and numbers of the antisense oligoprobes used for detection of the different (pre-) rRNAs by Northern Blotting are indicated with bars. (**B**) shows the pathway(s) of pre-rRNA processing and the sub cellular location of the respective rRNA precursors. Processing events are written in blue. Processing intermediates are classified as “early”, “intermediate” or “late” on the right. Early processing events in the 5’ ETS and the ITS1 (at or around site A_2_) can already occur co-transcriptionaly in *S. cerevisiae*. The latest processing steps that are inhibited after *in vivo* depletion of selected r-proteins or biogenesis factors (as indicated by the accumulation of the (pre-) rRNA intermediate(s) upstream of the inhibited step) are indicated in red.
(PDF)

Figure S2 Yeast strains used in this study. (XLSX)

Figure S3 Plasmids used in this study. (XLSX)

Figure S4 Oligonucleotides used in this study. (XLSX)

Figure S5 Analyses of (pre-) rRNAs co-purifying with selected FLAG tagged LSU r-proteins. Cellular extracts of 16 yeast strains each of which ectopically expressing a FLAG tagged version of a LSU r-protein complementing the corresponding lethal gene deletion(s) were subjected to affinity purification using an anti-FLAG matrix as described in Materials and Methods. An untagged wild type yeast strain and a yeast strain expressing a FLAG-tagged version of the Ubiquitin moiety of the Ubiquitin-rpL40A fusion protein (“FLAG-Ubq*”) were included in the analyses. The (pre-) rRNA content of the total cellular extracts (“Input” lanes 1-18) or of parts of the affinity purified fractions (“IP” lanes 19-36) were analysed by Northern Blotting using the indicated probes. A fraction of the cellular extract from an untagged yeast strain (lane 37) was used as reference to enable quantification of the relative amounts of the co-purified (pre-) rRNAs. The procedure was performed using two different concentrations of potassium chloride. The lysis buffers of the affinity purifications shown in (**A**) and (**B**) contained 200mM and 300mM potassium chloride, respectively. Equal signal intensities of the reference wild type Input and each IP fraction correspond to 1% co-purification efficiencies. The quanitifications shown in Figure 4 are derived from two reproduced northern blots of the same affinity purications for each concentration of potassium chloride. The average (pre-) rRNA co-purification efficiencies of the 16 FLAG-tagged rpLs shown in Figure 4A exclude the untagged wild type (lane 19) and the FLAG-Ubq* (lane 36) strains. The generation time of each yeast strain in YPD was determined as described in Materials and Methods and is listed in Figure S2.
(PDF)

Figure S6 Impact of *in vivo* depletion of rpL21 or rpL43 on association of FLAG tagged rpL2 and rpL3 with 7S pre-rRNA containing LSU precursors. Yeast strains which ectopically express either rpL43 (Y1103) or rpL21 (Y1100) under the control of the GAL1/10 promoter and a wild type yeast strain (Y207) were transformed with plasmids coding for a FLAG tagged version of rpL2 (TK1028) or rpL3 (TK1029) under control of the RPS28 promoter. Transformants were cultivated in galactose-containing medium and shifted for 4 hours to glucose containing medium to shut down the expression of the respective r-protein gene. Cellular extracts of these strains were subjected to affinity purification using an anti-FLAG matrix as described in Materials and Methods. (**A**) the (pre-) rRNA content of the total cellular extracts (“Input” lanes 1-3) or of parts of the affinity purified fractions (“IP” lanes 4-6) was analysed by northern blotting using the indicated probes. Changes in co-purification efficiencies of the 7S pre-rRNA after shutting down the expression of RPL43 or RPL21 were quantified in (**B**) in relation to the amount of 7S pre-rRNA co-purified in the reference wild type strain. Relative amounts of the 7S pre-rRNA co-purified via rpL2-FLAG and rpL3-FLAG are shown in dark and light grey, respectively. Quantification was performed from two biological replicates. The standard deviations are indicated as error bars.
(PDF)

Figure S7 (Pre-) rRNA composition of different preribosomal particle populations affinity purified after*in vivo* depletion of rpL21. The indicated derivates of a wild type yeast strain and of a strain in which rpL21 expression is under control of the GAL1/10 promoter were created which chromosomally encode TAP-tagged version of the LSU biogenesis factors Nog1, Nog2, Rsa4, Nop53 or Nop7. Strains were cultivated for four hours in glucose-containing medium to shut down (or not) expression of RPL21. The TAP-tagged proteins and associated pre-ribosomal particles were then affinity purified from corresponding cellular extracts as described in Materials and Methods. In (**A**) are shown the relative amounts of 5.8S rRNA and 7S pre-rRNA in the affinity purified fractions as detected by total RNA extraction and northern blotting using the indicated probe which is complementary to 5.8 rRNA sequences. Lanes 1-3 (using tagged Nog1, Nog2, and Rsa4, respectively, are derived from the experiments shown in Figures 6, 8, and 9, respectively. In (**B**) the (pre-) rRNA content of total cellular extracts (Input lanes 1-5) or fractions (IP lanes 6-10) affinity purified via Nop53-TAP or Nop7-TAP from cells expressing or not rpL21 are shown. Detected (pre-) rRNAs are indicated on the left and oligonucleotides used for (pre-) rRNA detection are indicated on the right. Purification efficiencies of the bait proteins were monitored by western blotting (see panel designated WB bait). (**C**) Shows a quantitation of the average co-purifications efficiencies of 5.8S rRNA and 7S pre-rRNAs with the indicated tagged LSU biogenesis factors in presence or upon depletion of rpL21 seen in two experiments. The scale for the 7S pre-rRNA co-purification efficiency is on the left side, the one for the 5.8S rRNA is on the right side. The internal background level of the experiments, as measured by the efficiency of 20S pre-rRNA co-purification, is indicated by a red line. Scale for the internal background is the one of 5.8S rRNA on the right.
(PDF)
