# Supplementary figures and images for: Studies on the Assembly Characteristics of Large Subunit Ribosomal Proteins in S. cerevisae
Source: PLoS One. 2013 Jul 10;8(7):e68412. doi: 10.1371/journal.pone.0068412 (PMC3707915; doi:10.1371/journal.pone.0068412)

**A**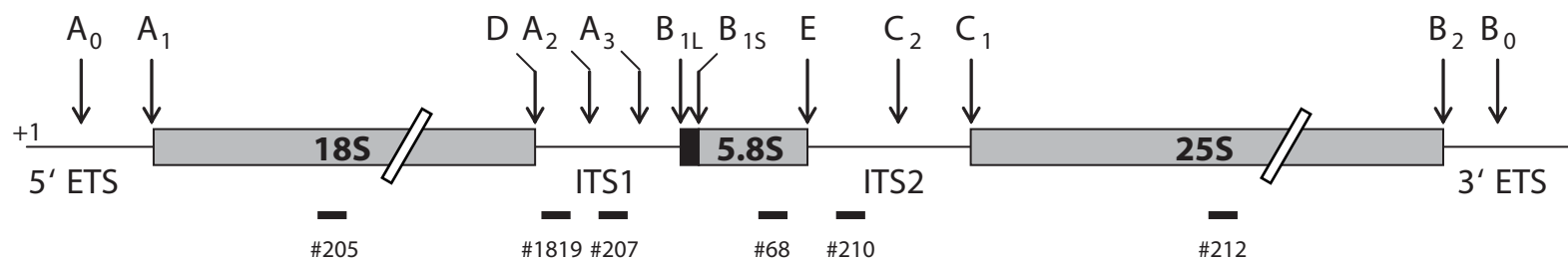**B**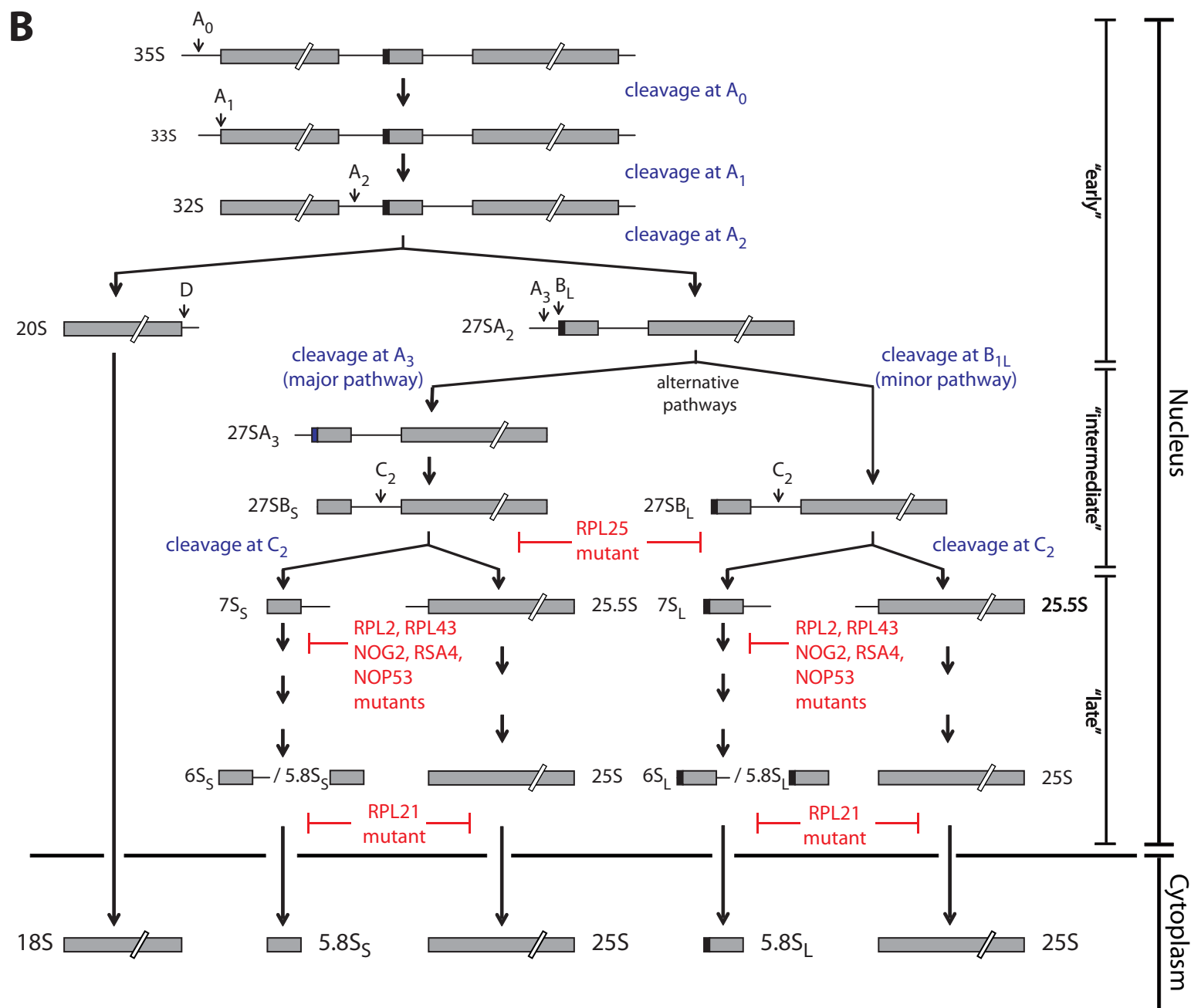

Supplement: Figure S1 — Three of the four rRNAs found in the mature ribosome are derived from the polycistronic transcript (A) made by RNA polymerase I which is processed through a series of endo- and exonucleolytic reactions. (A) illustrates the transcription start site (+1), the external transcribed spacer regions (5’ ETS1, 3’ ETS), the internal transcribed spacer regions (ITS1, ITS2), and the major pre-rRNA processing sites. The sizes of the indicated regions are not in proportion to their real length. The positions and numbers of the antisense oligoprobes used for detection of the different (pre-) rRNAs by Northern Blotting are indicated with bars. (B) shows the pathway(s) of pre-rRNA processing and the sub cellular location of the respective rRNA precursors. Processing events are written in blue. Processing intermediates are classified as “early”, “intermediate” or “late” on the right. Early processing events in the 5’ ETS and the ITS1 (at or around site A2) can already occur co-transcriptionaly in S. cerevisiae. The latest processing steps that are inhibited after in vivo depletion of selected r-proteins or biogenesis factors (as indicated by the accumulation of the (pre-) rRNA intermediate(s) upstream of the inhibited step) are indicated in red. (PDF) [file pone.0068412.s001.pdf]

**A**

## FLAG IPs of 16 rpLs (+untagged + Ubq\*) 200mM KCl

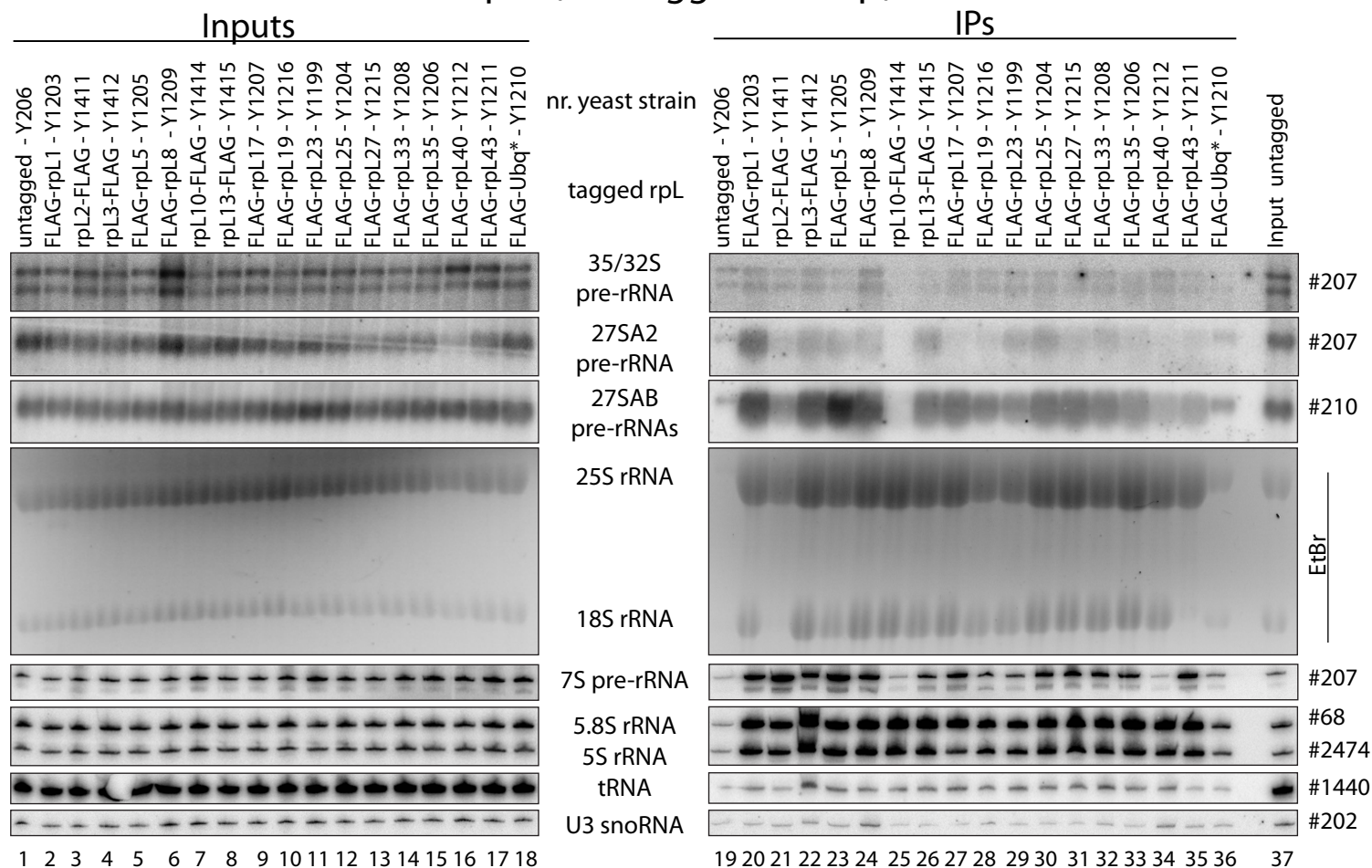**B**

## FLAG IPs of 16 rpLs (+untagged + Ubq\*) 300mM KCl

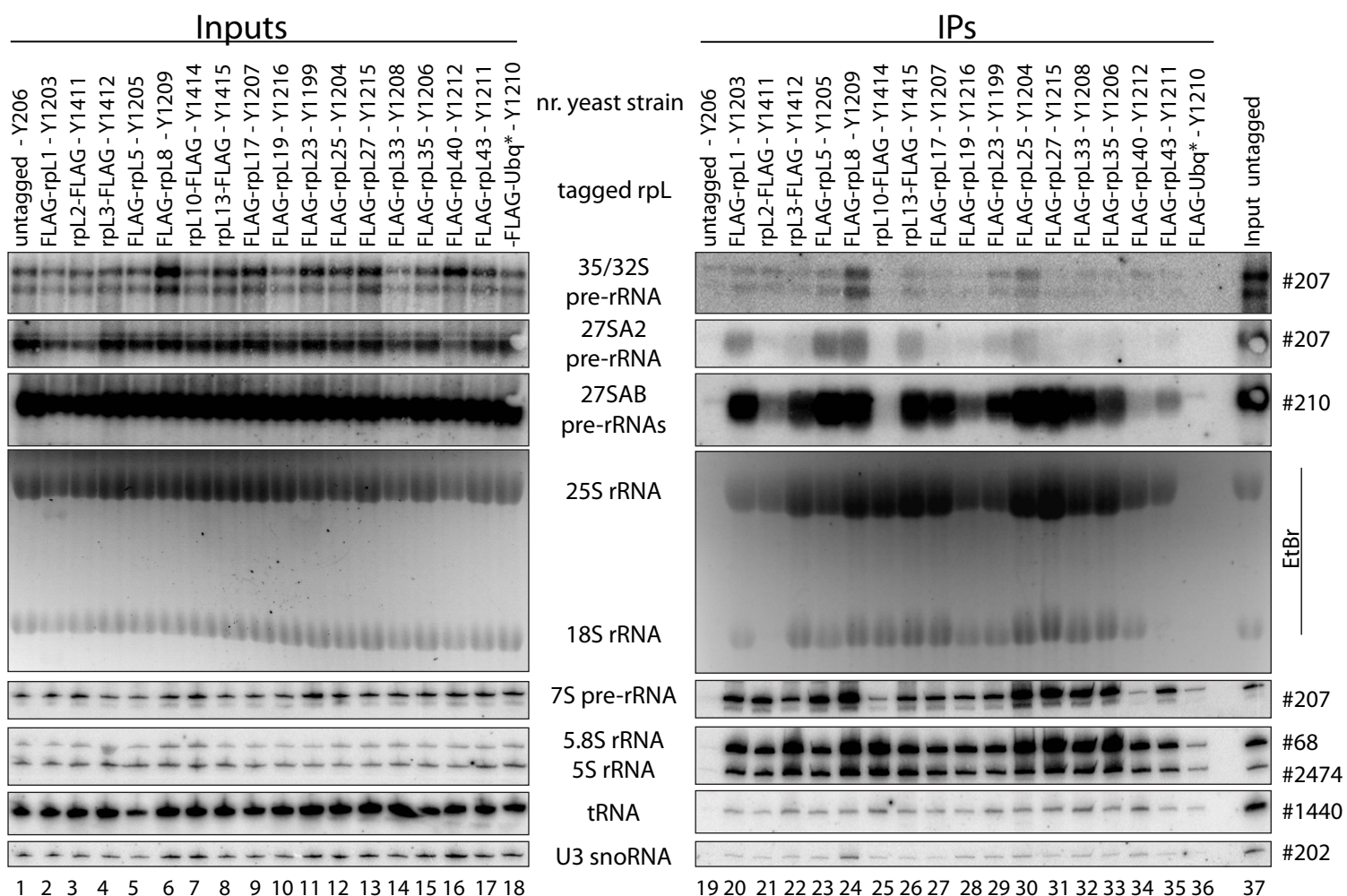

Supplement: Figure S5 — Cellular extracts of 16 yeast strains each of which ectopically expressing a FLAG tagged version of a LSU r-protein complementing the corresponding lethal gene deletion(s) were subjected to affinity purification using an anti-FLAG matrix as described in Materials and Methods. An untagged wild type yeast strain and a yeast strain expressing a FLAG-tagged version of the Ubiquitin moiety of the Ubiquitin-rpL40A fusion protein (“FLAG-Ubq*”) were included in the analyses. The (pre-) rRNA content of the total cellular extracts (“Input” lanes 1-18) or of parts of the affinity purified fractions (“IP” lanes 19-36) were analysed by Northern Blotting using the indicated probes. A fraction of the cellular extract from an untagged yeast strain (lane 37) was used as reference to enable quantification of the relative amounts of the co-purified (pre-) rRNAs. The procedure was performed using two different concentrations of potassium chloride. The lysis buffers of the affinity purifications shown in (A) and (B) contained 200mM and 300mM potassium chloride, respectively. Equal signal intensities of the reference wild type Input and each IP fraction correspond to 1% co-purification efficiencies. The quanitifications shown in Figure 4 are derived from two reproduced northern blots of the same affinity purications for each concentration of potassium chloride. The average (pre-) rRNA co-purification efficiencies of the 16 FLAG-tagged rpLs shown in Figure 4A exclude the untagged wild type (lane 19) and the FLAG-Ubq* (lane 36) strains. The generation time of each yeast strain in YPD was determined as described in Materials and Methods and is listed in Figure S2. (PDF) [file pone.0068412.s005.pdf]

**A**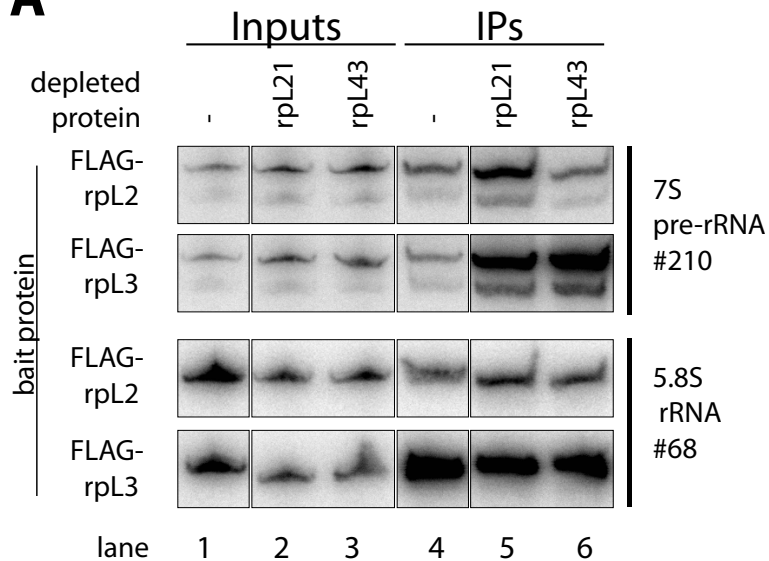**B**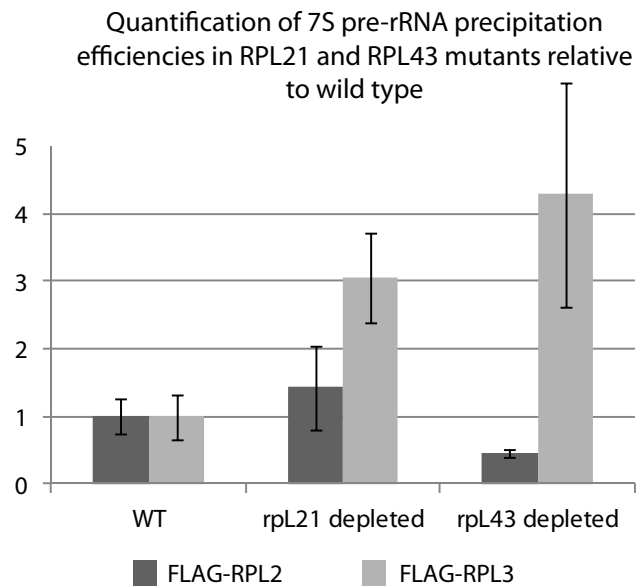

Supplement: Figure S6 — Yeast strains which ectopically express either rpL43 (Y1103) or rpL21 (Y1100) under the control of the GAL1/10 promoter and a wild type yeast strain (Y207) were transformed with plasmids coding for a FLAG tagged version of rpL2 (TK1028) or rpL3 (TK1029) under control of the RPS28 promoter. Transformants were cultivated in galactose-containing medium and shifted for 4 hours to glucose containing medium to shut down the expression of the respective r-protein gene. Cellular extracts of these strains were subjected to affinity purification using an anti-FLAG matrix as described in Materials and Methods. (A) the (pre-) rRNA content of the total cellular extracts (“Input” lanes 1-3) or of parts of the affinity purified fractions (“IP” lanes 4-6) was analysed by northern blotting using the indicated probes. Changes in co-purification efficiencies of the 7S pre-rRNA after shutting down the expression of RPL43 or RPL21 were quantified in (B) in relation to the amount of 7S pre-rRNA co-purified in the reference wild type strain. Relative amounts of the 7S pre-rRNA co-purified via rpL2-FLAG and rpL3-FLAG are shown in dark and light grey, respectively. Quantification was performed from two biological replicates. The standard deviations are indicated as error bars. (PDF) [file pone.0068412.s006.pdf]

**A**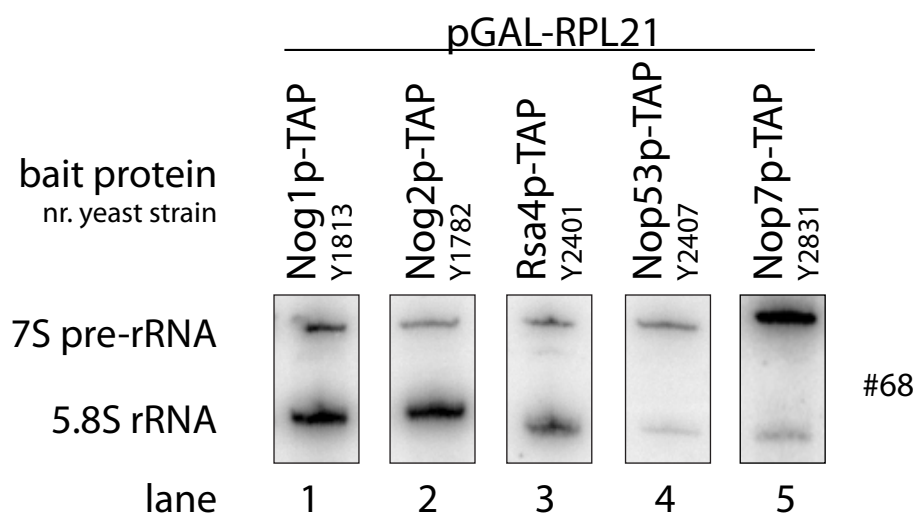**B**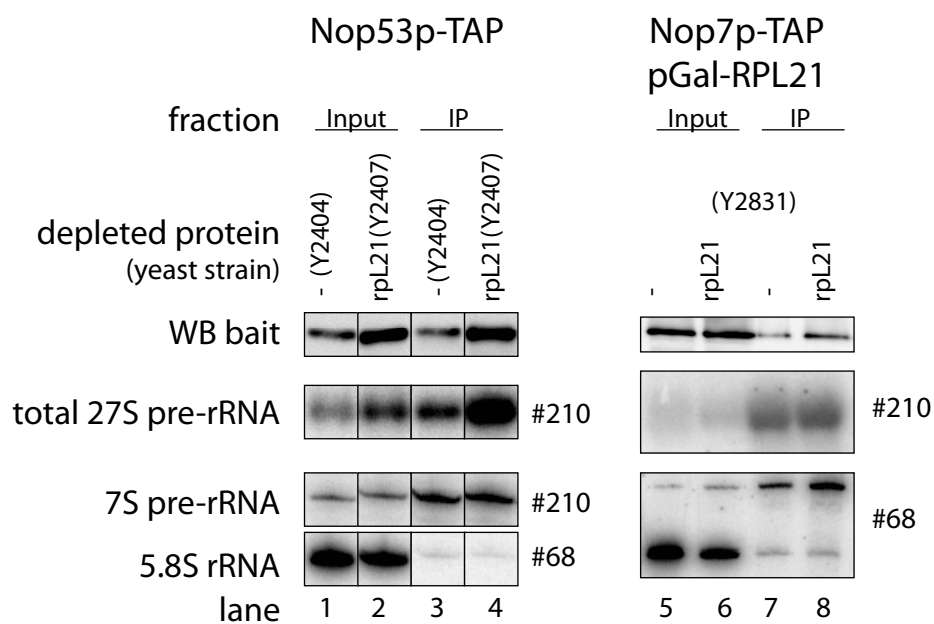**C**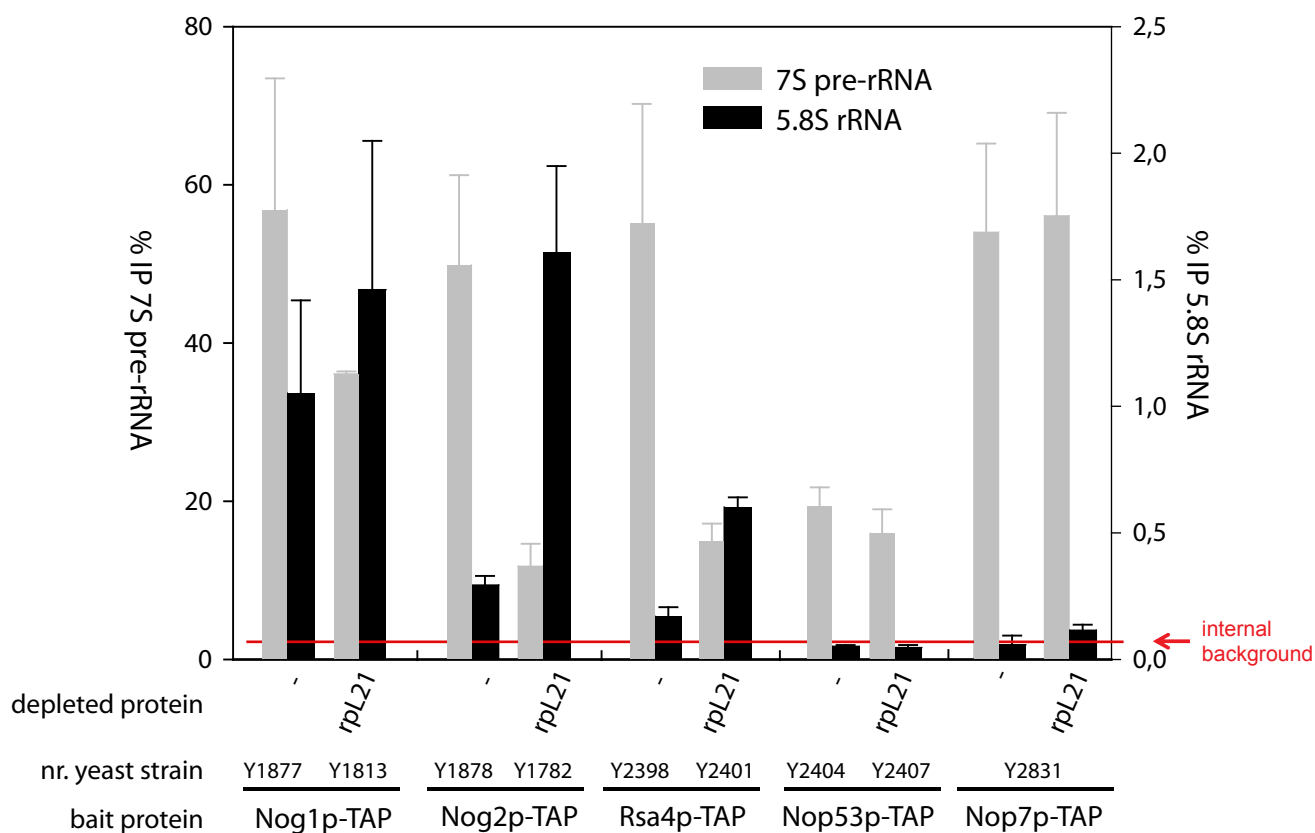

Supplement: Figure S7 — The indicated derivates of a wild type yeast strain and of a strain in which rpL21 expression is under control of the GAL1/10 promoter were created which chromosomally encode TAP-tagged version of the LSU biogenesis factors Nog1, Nog2, Rsa4, Nop53 or Nop7. Strains were cultivated for four hours in glucose-containing medium to shut down (or not) expression of RPL21. The TAP-tagged proteins and associated pre-ribosomal particles were then affinity purified from corresponding cellular extracts as described in Materials and Methods. In (A) are shown the relative amounts of 5.8S rRNA and 7S pre-rRNA in the affinity purified fractions as detected by total RNA extraction and northern blotting using the indicated probe which is complementary to 5.8 rRNA sequences. Lanes 1-3 (using tagged Nog1, Nog2, and Rsa4, respectively, are derived from the experiments shown in Figures 6, 8, and 9, respectively. In (B) the (pre-) rRNA content of total cellular extracts (Input lanes 1-5) or fractions (IP lanes 6-10) affinity purified via Nop53-TAP or Nop7-TAP from cells expressing or not rpL21 are shown. Detected (pre-) rRNAs are indicated on the left and oligonucleotides used for (pre-) rRNA detection are indicated on the right. Purification efficiencies of the bait proteins were monitored by western blotting (see panel designated WB bait). (C) Shows a quantitation of the average co-purifications efficiencies of 5.8S rRNA and 7S pre-rRNAs with the indicated tagged LSU biogenesis factors in presence or upon depletion of rpL21 seen in two experiments. The scale for the 7S pre-rRNA co-purification efficiency is on the left side, the one for the 5.8S rRNA is on the right side. The internal background level of the experiments, as measured by the efficiency of 20S pre-rRNA co-purification, is indicated by a red line. Scale for the internal background is the one of 5.8S rRNA on the right. (PDF) [file pone.0068412.s007.pdf]
